# Barriers and Facilitators to The Involvement of Under-Represented Children and Young People (aged 8–25) in Mental Health Research – a Systematic Review

**DOI:** 10.1007/s10567-025-00544-4

**Published:** 2025-09-22

**Authors:** Rachel Perowne, Sarah Rowe, Azin Lajevardi, Luke Bingham, Ella Parry, Gabrielle Grey, Pamela Carien Thomas, Leslie Morrison Gutman

**Affiliations:** 1 UCL Centre for Behaviour Change, Division of Psychology and Language Sciences, University College London, London, UK; 2 Division of Psychiatry, University College London, London, UK; 3 Department of Clinical, Educational and Health Psychology, University College London, London, UK

**Keywords:** Mental health research, Young people, Children and adolescents, Patient and Public Involvement, Under-representation, Diversity, Behaviour Change Wheel

## Abstract

Young people’s involvement in mental health research enhances its relevance and impact. Involvement means conducting research *with* young people rather than just *about* them. However, under-representation persists among certain groups of young people, including ethnic minorities, those with disabilities, from lower-income households, with immigrant backgrounds and young people communicating in a language other than their first language. This systematic review applies the Behaviour Change Wheel (BCW) to explore barriers and facilitators to involving under-represented young people in mental health research and identify potential strategies for improvement. Five electronic databases and grey literature were systematically searched. Included studies focussed on children and young people (aged 8–25) from under-represented backgrounds involved in mental health research. The BCW was used to categorise barriers and facilitators as well as to map strategies to Intervention Functions. Five young people formed an oversight group, with three serving as co-researchers, contributing to search design, screening, data extraction and discussion. Twenty-seven studies from seven countries met the inclusion criteria. The majority of barriers and facilitators were linked to physical and social opportunity. Digital exclusion, time constraints, bureaucratic hurdles and mistrust of researchers were all barriers. Facilitators included flexible structures, inclusive communication and relationship building. Reported strategies to improve involvement aligned with enablement and environmental restructuring. Inclusive, adaptive research practices and improved reporting are needed to meaningfully engage under-represented young people in mental health research. Future research should prioritise perspectives of under-represented young people to better understand and address the barriers they face.

**Supplementary Information:**

The online version contains supplementary material available at 10.1007/s10567-025-00544-4.

## Introduction

The reported increase in young people’s mental health issues is a serious public health concern in the UK (Grimm et al., 2022). Adding to this concern is the significant number of young people who do not access professional help or support for their mental health. Reasons include young people’s negative beliefs about mental health services and professionals, stigma and the failure of services to meet the needs of young people, in both capacity and design (Aguirre Velasco et al., 2020; Appleton et al., 2022; Punton et al., 2022). Certain groups of young people may also be under-served because of specific barriers to service engagement, such as cultural taboos for young people of certain ethnic or cultural backgrounds (Coelho et al., 2022), fear of unfamiliar environments for refugee youth (Fazel et al., 2016), fear of harassment for LGBTQIA + young people and service inaccessibility for those living in rural areas (Brown et al., 2016).

It is widely accepted that research involving people with lived experience, for example through Patient and Public Involvement (PPI), is more robust and impactful (Shimmin et al., 2017; Watson et al., 2023) and services which involve young people in their design are more successful (Meeting the Needs of Young Adults within Models of Mental Health Care, 2022). Involvement can take many forms; including PPI, co-design and different models of participatory research, such as Community-Based Participatory Research and Participatory Action Research, to name a few. There are numerous definitions of involvement including the established National Institute of Health and Care Research (NIHR) definition of “research being carried out ‘with’ or ‘by’ members of the public rather than ‘to’, ‘about’ or ‘for’ them”(Simons, 2012) as well as more detailed, and recent, definitions such as “community partners actively collaborat(ing) in the governance, priority setting and conduct of research, and in sharing and applying its resulting knowledge” (MacSweeney et al., 2019).

Improving diversity within involvement ensures that all groups have a voice on issues that affect them and gives those who have been marginalised the opportunity to shape services to meet their needs (Reynolds et al., 2021). However, critics suggest that PPI is dominated by “the usual suspects” and that there has been a failure to make involvement open to all (Rouncefield-Swales et al., 2021). In young people’s health research, reporting varies, but certain groups, such as ethnic minorities and those with complex needs, are often under-represented and those who do get involved tend to be confident, outgoing, and comfortable in formal education settings (Reynolds et al., 2021; Rouncefield-Swales et al., 2021). This has implications for the effectiveness and generalisability of research because those who are most affected by inequalities are often the least likely to be involved in research (Reynolds et al., 2021). This is echoed in youth mental health research (Michail, 2024), where expert consensus highlights poor diversity and under-representation of young people from ethnic minority backgrounds, with disabilities or complex health needs, from low-income households, migrants and those with refugee or asylum seeker status and for whom English is not a first language (Perowne et al., 2024). To address this inequality, we need to understand the barriers to involvement in mental health research for under-represented young people, in order to develop strategies to overcome them and help researchers involve a more diverse range of young people.

Existing research exploring barriers and facilitators to under-represented young people’s involvement in mental health research is limited. Some of the previously reported barriers to diversity in involvement across health and social care research include: those from outside academia not considering that their contributions will be valued; the capacity for those with certain health conditions to be involved varying day-to-day; lack of sufficient financial incentives, or delays in receiving them, meaning that those from lower socio-economic groups cannot afford to be involved (Simons, 2012). Additionally, concern around stigma can be a particular barrier to involvement for those with mental health issues (Shimmin et al., 2017).

A small number of reviews have explored young people’s involvement in mental health research (e.g. McCabe et al., 2023). Barriers and facilitators included relational factors such as power dynamics, and process factors such as time and methodology. However, these reviews have not focussed on barriers and facilitators to under-represented young people or been underpinned by theory. Application of theory provides an evidence-based framework around which to structure findings, offers an opportunity to test the validity of the theory and can ultimately lead to better outcomes (Hayes et al., 2023).

**Fig. 1:**
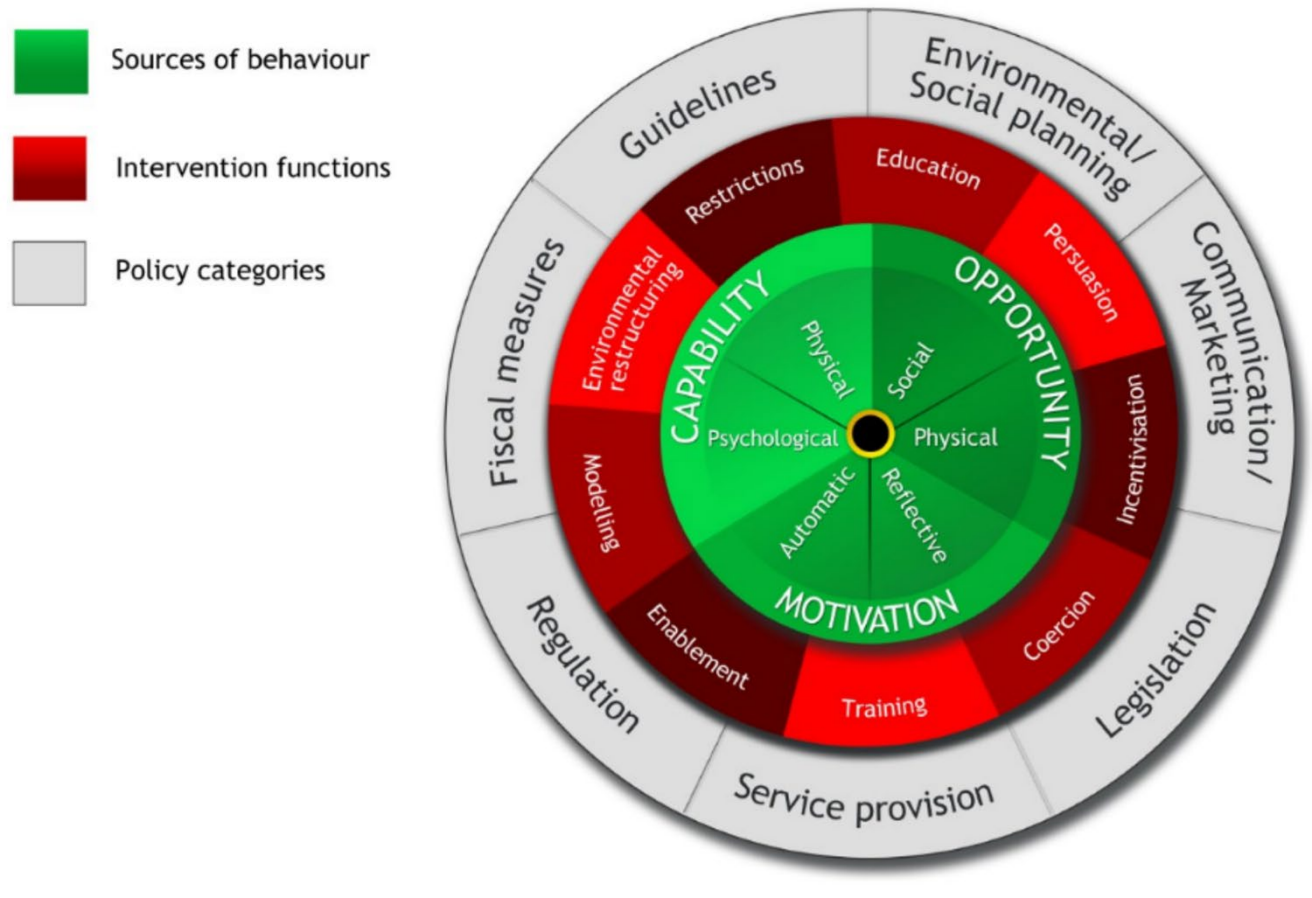
The Behaviour Change Wheel (Michie et al., 2014)

The current systematic review applies the Capability, Opportunity, Motivation and Behaviour (COM-B) model (Michie et al., 2011) to analyse, and categorise, barriers and facilitators to involvement. In the COM-B model, behaviour is influenced by motivation (which can be reflective or automatic), opportunity (physical or social) and capability (physical or psychological). Opportunity and capability can act directly to influence the behaviour, or via motivation (Michie et al., 2011). The COM-B model is particularly valuable as it sits at the core of the Behaviour Change Wheel (BCW) (see Fig. 1), an integrated framework, developed through the synthesis of 19 key behaviour change frameworks (Michie et al., 2014). It provides evidence-based linkages between the COM-B categorised influences on behaviour and nine broad “Intervention Functions” which can be used to change behaviour. These Intervention Functions include education, training, persuasion, incentivisation, coercion, restriction, modelling, environmental restructuring and enablement. Used together, the COM-B model and BCW allow both the identification and categorisation of barriers and facilitators and the corresponding intervention strategies which are, according to expert consensus, most likely to be effective for behaviour change. The BCW has been used successfully in other reviews in a health context, for example to understand the behavioural influences targeted in children’s obesity interventions (Johnson et al., 2018) and to identify and categorise the Intervention Functions present in interventions to facilitate shared decision making in youth mental health (Hayes et al., 2023).

Research questions for this systematic review are:

- What is the empirical research focussed on barriers and facilitators to involving under-represented children and young people (aged 8–25) in mental health research?
- What are the barriers and facilitators to the involvement of under-represented children and young people (aged 8–25) in mental health research?
- Are there differences in barriers and facilitators experienced by under-represented children and young people with regard to their different demographic characteristics?
- What strategies have been identified to overcome these barriers?

## Methods

### Search Strategy

This systematic review was conducted according to the Preferred Reporting Items for Systematic reviews and Meta-Analyses Statement (PRISMA) guidance for conducting and reporting systematic reviews (Page et al., 2021) and the completed PRISMA checklist is available in the supplementary materials. The protocol for the study was registered in advance with the PROSPERO (International Prospective Register of Systematic Reviews) database (registration number CRD42024523551).

Five relevant scientific databases were searched: MEDLINE, Embase, PsycINFO, CINAHL and Web of Science. Searches of all the databases were conducted on 18 March 2024 and rerun on 16 March 2025, to capture papers published in the intervening period. Grey literature was searched using PsycEXTRA, Google Scholar and Google to capture dissertations, theses, government or National Health Service (NHS) (or equivalent) reports. Two young co-researchers (EP and AL) contributed to the grey literature searches using Google and Google Scholar. Additional studies were also sought through handsearching the reference lists of other relevant systematic reviews, as well as forward and backward referencing of included studies. Searches were limited to English language, and studies conducted in OECD countries, which have broadly similar research environments and are therefore likely to include findings that are more relevant to the UK context (Deckert et al., 2024; National Institute for Health & Clinical Excellence, 2018). For comprehensiveness, no time limits were applied. The concepts included were developed using the PICO structure and included, “children and young people”, “mental health”, “involvement” and “research”. A combination of subject headings (or “MeSH” terms) and key words were searched. Children and young people included those between the ages of 8 and 25 years, in line with the definition in McCabe et al. (2023).

The NIHR definition of “involvement” (Simons, 2012) was used to guide the search. However, a large number of terms are utilised in the literature for involvement, from methodological terms such as “Participatory Action Research” to specific mechanisms for involvement such as “Young People’s Advisory Groups”. An extensive list of these terms was searched, guided by terms included in other similar systematic reviews (for example, McCabe et al., 2023; Nowland et al., 2022; Sales et al., 2021), as well as other recent research on this topic (Perowne et al., 2024), in an effort to be comprehensive. Two young co-researchers (EP and LB) reviewed the proposed search terms and provided feedback on their relevance. Advice from a specialist librarian was also incorporated. Synonym terms were combined using the OR Boolean operator and concepts combined using the AND operator. The full searches from each database are available in the supplementary materials.

### Eligibility Criteria

All types of primary study design were included: qualitative, mixed method and quantitative. Studies were included both where young people’s involvement was the primary focus and where it was reported alongside a different primary aim. Studies where young people were participants, or passively involved, were excluded as, for the purposes of this study, this did not constitute involvement (Perowne et al., 2024). Under-represented groups included ethnicity, socio-economic status, first language, immigration status (immigrant, asylum seeker, refugee), disability or complex health needs (Perowne et al., 2024), as well as age and sex/gender (Reynolds et al., 2021; Rouncefield-Swales et al., 2021). Table 1 provides a summary of the inclusion and exclusion criteria for the title and abstract review stage.

**Table 1:** Inclusion and exclusion criteria

Inclusion	Exclusion
Qualitative, mixed methods, quantitative studies such as randomised controlled trials, surveys, cross sectional studies Systematic reviews, meta-analyses and protocols which involve children and young people in the review process Studies published any time Studies published in English Studies conducted in OECD countries	Conference abstracts, literature reviews, commentaries and opinion pieces such as letters or comments Systematic reviews, meta-analyses and protocols which did not involve children and young people in the review process Studies in languages other than English Studies conducted outside the OECD
Mental health, mental health disorders in children and young people such as anxiety, depression, eating disorders, self-injury/self-harm and suicidal ideation Research including clinical research such as trials, research in to health services and support, community-based research, health policy research, health promotion research Involvement of young people in Participatory Action Research, Young People’s Advisory Groups, Patient and Public Involvement, co-production activities, young people as peer/co-researchers, advisors or consultants	Mental wellbeing or positive mental health such as mindfulness. Psychosocial problems (e.g. bullying, adverse childhood experiences and substance misuse). Neurodevelopmental disorders such as Autism Spectrum Disorder and Attention Deficit Hyperactivity Disorder. Mental health of parents and carers Young people as participants or the recipients of research outputs e.g. through dissemination activities or involvement of parents/carers/other adults as a proxy for children and young people
Involvement of children and young people aged between 8 and 25 Perspectives of children and young people, researchers, PPI professionals or others within the research process	Involvement of children and young people under 8 or over 25 (or where less than 75% of those involved were between these ages) Perspectives of others who have not been involved with the process of involving young people in research

## Study Selection

Titles and abstracts were exported into EndNote 20 for deduplication and then into Covidence for screening. All titles and abstracts were screened by one reviewer (RP), with 20% independently screened by a second reviewer (either PT, LB or AL). Results were compared, with 85% agreement. Discrepancies were discussed until full agreement was reached. Full texts were screened by one reviewer (RP) and 20% were independently screened by a second reviewer (either GG or AL) using the pre-determined inclusion/exclusion criteria, with 84% agreement. Discrepancies were discussed until full agreement was reached. RP reviewed the remaining studies for inclusion.

## Data Extraction

Data were extracted by the lead author (RP) using a pre-defined data extraction table. As data relating to involvement was not confined to a specific section of the papers, full texts were examined in detail to identify and extract relevant information. A second reviewer (LB) independently completed data extraction on five papers. This was then cross-checked (by RP) to ensure consistency before data from the remaining studies were extracted. The data extracted included: title, authors, data source (e.g. journal title), country, year of publication, research aims, design and setting, details of the young people’s involvement (such as the framework utilised, stages of research in which young people were involved), training provided, demographics of the young people involved, main outcome of interest; barriers and facilitators reported to young people’s involvement in the research process, and any strategies to improve involvement suggested by the authors.

## Quality Appraisal

Given the focus of this systematic review, a quality appraisal tool was selected that assessed the quality of reporting of involvement activities rather than the quality of the study itself. The Quality of Reporting Involvement and Engagement of Patients and the Public Appraisal Tool (QRIPPAT) was used (Rouncefield-Swales et al., 2021). This novel tool was developed to address the lack of appropriate framework for appraising the quality of PPI reporting across multiple studies. It is based on the core aspects of the validated GRIPP2 checklist (Staniszewska et al., 2017). It includes ten questions organised under five main GRIPP2 themes: aims and definition of PPI, methods of PPI, findings and discussion and finally, learnings and reflection of the PPI. In line with Rouncefield-Swales et al. (2021), a matrix was created to indicate which of each of the ten criteria were met by each study (using a “yes” (one point), “no” (zero points) or “somewhat” (half a point) rating). An overall rating was calculated (by RP) for each study from zero—low quality reporting, to ten—high quality reporting (Rouncefield-Swales et al., 2021).

## Data Synthesis and Analysis

Extracted data on barriers and facilitators was exported into an Excel spreadsheet and analysed using a two-stage thematic analysis (Braun & Clarke, 2006). An inductive approach was used initially to generate codes, which were then compared and grouped into overarching themes. These themes were then analysed deductively by mapping them onto components of the COM-B model of behaviour (McDonagh et al., 2018; Michie et al., 2014). These were further analysed for differences between groups including ethnicity, socio-economic status, first language, immigration status (immigrant, asylum seeker, refugee), disability or complex health needs, as well as age and sex/gender.

Following a similar process, strategies to optimise the involvement of under-represented young people extracted from the included studies were first coded inductively to identify themes. These were then coded deductively to categorise each strategy according to the Intervention Functions of the Behaviour Change Wheel (Michie et al., 2014).

A count of the number of studies reporting each theme in both syntheses was recorded, and these are presented as part of the results.

## Young People’s Involvement in This Study

A group of diverse young people, identified from within an existing NIHR Young People’s Advisory Group, was involved throughout this systematic review. Five young people formed a “Young Researchers’ Oversight Group” (YROG), advising on the review throughout the project, from agreeing the research question to providing their comments on the findings and final report. Meetings took place throughout the study, roughly every two months via Zoom. The young people were between the ages of 16 and 18 years. Two identified as male and three as female. White, Asian British, Arab and “multiple ethnic groups” were represented. World views included Christian, Muslim and Atheist or Agnostic. Sexual orientations included heterosexual, gay and questioning. Levels of parental education ranged from GCSE to Doctoral degree (or equivalents) and four of the five young people were in full‐time education. All five were born in the United Kingdom, but four had a parent born outside the United Kingdom and for one, English was not their first language. The group included young people who had experienced mental health problems, disability or long‐term health conditions and who were neurodiverse. One had caring responsibilities. The young people lived in a variety of locations in England: urban, suburban, and rural.

Three of these young people (AL, EP and LB) were involved as co-researchers, contributing to the study by suggesting terms for the search strategy as well as searching grey literature. They helped to refine the inclusion and exclusion criteria, acted as second reviewers in screening and data extraction and provided their views on the results, which have been incorporated into the discussion section. Further reflections and evaluation of the young people’s involvement will be reported in detail elsewhere.

Young people were provided with training on all tasks they took part in and were compensated in line with NIHR guidelines (Payment Guidance for Researchers & Professionals, 2023). Due to the extensive involvement, the approximate cost of involvement activities was £1,500. The young people's involvement is reported in this paper using the GRIPP2 checklist (Staniszewska et al., 2017) for the transparent and consistent reporting of PPI activities in research (available in the supplementary materials).

**Fig. 2:**
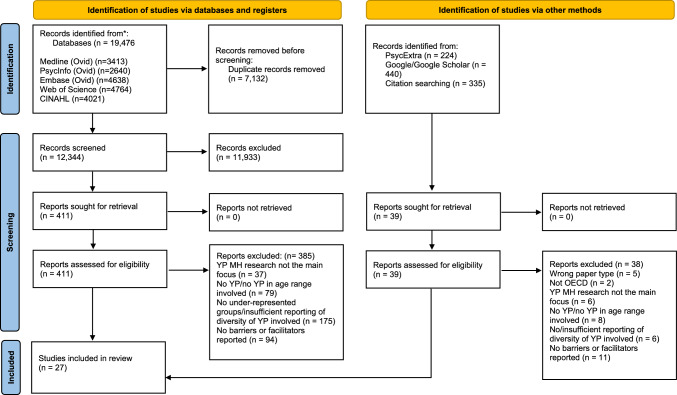
PRISMA flow diagram

**Results**The PRISMA diagram for the search and selection is shown in Fig. 2.

A total of 13,343 studies were identified across the initial and updated searches (after duplicates were removed) and the grey literature search. Agreement between the reviewers was over 80% for both stages of the screening and extraction. Full texts of 450 papers were reviewed and 27 studies were included in the analysis, representing the empirical research found which focussed on barriers and facilitators to involving under-represented children and young people (aged 8–25) in mental health research (RQ1). Four of the papers reported the same two studies but from different perspectives and therefore all four were included (Inge et al., 2024; Pérez-Aronsson et al., 2022; Povey et al., 2020, 2022).

## Description of Studies

Papers were published from seven countries: Australia (n = 8), Canada (n = 6), Netherlands (n = 1), Norway (n = 1), Sweden (n = 2), UK (n = 4) and US (n = 5). They were published between 2010 and 2025, with two thirds being published after 2020 (n = 18). In 12 papers, involvement of young people was a key focus of the paper, in 13, descriptions of involvement activities were embedded in the report of the research project and the remaining two papers had a dual focus. Twelve studies were set in a health or mental health service context, seven in a community context (e.g. community organisations), four in an education setting and the remaining studies were either based in a combination or did not clearly report the setting. A table providing detailed descriptions of each study can be found in the supplementary materials.

## Young People’s Involvement in the Included Studies

### Numbers of Young People Involved

Table 2 summarises details of the young people’s involvement. Overall, over 520 young people were involved in the studies with an average (mean) of almost 20 young people per study. Co-design studies generally involved more young people, ranging from eight (Hetrick et al., 2018) to 75 (Cheng et al., 2021). In contrast, in studies where young people played an advisory or co-researcher role, the number of young people involved ranged from three (Inge et al., 2024) to 20 (Randall, 2018; Thomson et al., 2022).

**Table 2:** Involvement details

First Author, Year	Involvement methodology	Definition or explanation of framework/model?	Stages of research involvement	Involvement type/ Methods	Training?	Number of CYP	Age	Ethnicity/ racial/cultural group	Sex/ Gender	Other demographics
Anang et al. (2019)	CBPR, two-eyed seeing	Yes	Planning, Data collection	Regular meetings	Yes	12	High school students	Inuit	NR	NR
Babbage et al. (2024)	PPI and co-production, User Centred Design, Person-Based Approaches, Responsible Research and Innovation	Partial	Funding, planning, intervention development, dissemination	Interviews, focus groups, surveys, workshops	Yes	5 PPIs members, 11 in co-production workshop	PPI: 3 aged 21–26 others unknown, Co-production workshops between 17 and 25	PPI: 3 from minority ethnic groups, others unknown. Co-production workshops mix of White British, Black British, Asian Korean and Pakistani	PPI:3 female, others unknown Co-production workshops: male and female	PPI: 3 heterosexual, 1 long-term mental health condition, long-term physical health condition. Various levels of educational achievement
Blueprint Writng Collective.(2022)	PPI	Yes	Data collection, analysis, dissemination	Co-research (with a different group of children and young people also members of an advisory group)	Yes	6	18–25	NR	NR	All current undergraduates or recent graduates
Cheng et al. (2021)	Participatory design, Project Synergy Research and Development Synergy	Partial	Intervention development	10 × Co-design workshops	NR	75	19 aged 12–15, 56 age 16–25	9 Aboriginal or Torres Strait Islander people, 8 CALD people – included some adults	58 female, 44 male, 1 transgender, 2 gender neutral—included some adults	9 LGBTQIA + , 1 person with a disability (includes adults in count)
Culbong et al. (2023)	Aboriginal Participatory Action Research	Yes	Intervention development, dissemination	Co-design workshops, co-research	NR	3 CYP co-authored paper, 7 other co-researchers aged 16–25)	19, 29 (over age range but may not have been at time of study), unknown	Aboriginal	2 men, 1 woman	NR
Dosso et al. (2024)	NR	NR	Funding, planning, recruitment, knowledge translation	Advisory/ consultation and workshops	Yes	7	3 teenagers, 3 individuals in their 20s	NR	NR	2 with Autism Spectrum Disorder, 3 with physical health diagnoses. Many worked/studied in health/mental health-related field
Figueroa et al. (2025)	Participatory research	No	Intervention development, Analysis	Co-design and co-research	Yes	17	10 to 23	Dutch and Dutch/Moroccan, Dutch/Turkish, Dutch/ Arabic and Turkish	16 Female, 1 Male	NR
Hargrove, 2019	Participatory Action Research	Yes	Planning, implementation	Participant co-research	Yes	7	19–24	A range of Black ethnic backgrounds	5 cisgender women, 1 trans man, I cisgender man	5 heterosexual, 1 bisexual, 1 queer and pansexual. All were full time students. Socio-economic diversity
Hetrick et al. (2018)	Participatory Design/Youth Partnership	Yes	Intervention development	Co-design workshops	NR	8	18–25 years (mean 21.4)	NR	3 male, 8 female	NR
Inge et al. (2024)	PPI	Yes	Intervention development, data collection, dissemination	Co-research	NR	3	Target population was 15–24 years	Originated from Middle East and North Africa	1 young woman and 2 young men	All fluent in Swedish and had attended school there
Katapally, 2020	CBPR Participatory Action Research, two-eyed seeing, citizen science	Yes	Evaluation	Focus groups with Youth Citizen Scientist Council members	NR	76	13–18	Indigenous youth	Representation from various genders	Various socio-economic groups
Libon et al. (2023)	Participatory co-design	No	Design	3 × workshops	NR	11	15–24 (Median age 20)	NR	NR	Rural youth
Liegghio, 2013	Participatory Action Research	Yes	Planning, Design, Data Collection, Analysis	Weekly (then bi-weekly) meetings	Yes	7	1 14 year old, 3 15 year olds, 1 16 year old, 2 17 year olds	5 Caucasian, 1 Black/Caribbean, 1 bi-racial/Filipino and Middle Eastern	4 male, 3 female	1 bisexual, 6 heterosexual, 3 had family income under $30,000. Plus other diverse demographic backgrounds
Mance et al. (2010)	CBPR	Yes	Intervention development	Weekly consultation meetings	Yes	4	'Adolescents and young adults'	African American	2 male, 2 female	Low income urban communities
Perez-Aronsson et al. (2022)	Participatory	No	Intervention development	3 Workshops	NR	3	Youths	NR	Selection informed by… Gender	All migrants (Syria, Afghanistan and Somalia). Selection informed by arrival status (e.g. unaccompanied or accompanied)
Povey et al. (2022)	Participatory design	No	Design, Recruitment	5 or 6 co-design workshops and peer researchers	NR	45 (10 peer researchers)	10–18 years, Mean age 14.71	Aboriginal or Torres Strait Islander	24 Male and 21 Female	Linguistically diverse
Povey et al. (2020)	Participatory design	Yes	Design, Intervention development, review	Indigenous youth reference group (IYRG) (5 meetings) plus co-design meetings and workshops (16) with a separate group of CYP and young Indigenous researchers	NR	75	Co-design workshops—8–18 years, IYRG—15–25 years, mean age = 15.14	Indigenous/ Aboriginal or Torres Strait Islander descent	Female = 40, Male = 33, Gender diverse = 2	24 lived in very remote communities, 21 English not main language, 7 not engaged in school = 7
Randall, 2018	INVOLVE framework	Yes	NR	NR	NR	28	18–25	NR	Participants were predominately female (60.7%, n = 17)	Majority undergraduates (92.8%)
Rocha et al. (2023)	Youth-led Participatory Action Research	Yes	Design, survey development, recruitment, data management, analysis, interpretation and dissemination	YPAR/co-research	Yes	12 to 15	grade 6–12 (approx. 11–18 years)	Predominantly Latinx	NR	Predominantly immigrant youth
Savaglio et al. (2025)	Co-design and co-production	Yes for co-production	All stages including co-authoring	Co-design, supporting data analysis	NR	23	10 to 25	6 Aboriginal and/or Torres Strait Islander	11 female, 8 male, 4 Non-binary	NR
Schwartz et al. (2020)	Stakeholder-engaged approach	No	Intervention development, Data Analysis	Advisory board including 3 young people plus 3 young adult mental health/peer-mentoring research team (weekly meetings)	NR	6 in total (3 in YRT and 3 on advisory board)	average age 19.4	NR	YRT: 2 male and 1 female	3 with autism spectrum disorder, 3 with diverse intellectual/ developmental disabilities
Sheikhan et al. (2021)	McCain Model of Youth Engagement	Yes	Research design, intervention development, analysis, evaluation and implementation	Young People’s Advisory Group*	NR	22*	16–24*	NR	NR	NR
Standley, 2023	Youth GO	Yes	Data collection and analysis	Focus groups	Yes	10	14 to 18	6 white, 1 Slovak, 1 Indian, 1 Hispanic	5 male, 5 female/woman	8 straight, 2 bisexual
Stoyanov et al. (2021)	Participatory design	No	Intervention development	Co-design workshops	NR	25	7 × 12–15 years; 14 × 16–19 years, and 4 × 20–25 years	White, Asian, Middle Eastern, European	18 female, 7male	Education levels, Employment status, living arrangement, relationship status, receiving psychological treatment
Thomson et al. (2022)	PPI	Yes	Intervention development	Youth-led, co-production, advisory group	NR	20	21–25 but PPI café is 16–24	NR	NR	Majority white, middle‐class
Viksveen et al. (2022)	Not stated		Setting research priorities, funding applications, planning, data collection (mental health surveys, systematic review) and analysis, dissemination	Representatives and co-researchers	Yes	10	15 +		‘Both binary genders’	Varied cultural and health backgrounds
Walker et al. (2021)	INVOLVE framework to PPI	Yes	Analysis, dissemination	4 face to face meetings	NR	8	10–17 years	NR	3 male, assume other 5 were female but this is NR	Primarily neurological or rheumatic long-term conditions

*NR* not reported, *CYP* children and young people, *CALD* culturally and linguistically diverse, *PPI* patient and public involvement, *YPAR* youth participatory action research, *LGBTQIA* + lesbian, gay, bisexual, transgender, queer or questioning, intersex, asexual or aromantic, *YRT* young adult mental health/peer-mentoring research team

^*^data not reported in main paper but obtained from supplementary linked paper (Henderson et al., 2018)

## Demographics of The Young People Involved

The demographics reported of the young people involved was highly variable, with some studies providing detailed demographics at an individual level (e.g. Liegghio, 2013), others providing summary demographics at group level and a small number reporting very limited demographics. Of the 27 included papers, most (n = 22) reported at least the age range of those involved, with ages spanning 8 to 31 years. Nineteen studies made reference to the cultural background or ethnicity of the young people involved and in eight of the papers, a particular cultural or ethnic group was the target population for the study. Twenty studies reported on (or referenced) the sex/gender of the young people. Other demographic data included: education/employment status, health, physical or developmental disability or disorder, sexual orientation, first language, socio-economic status, urban/rural youth, refugee/migrant status. A number of studies acknowledged and commented on the lack of diversity of the young people involved.

## Features of Involvement

Almost all studies identified the model, methodology or framework of involvement used: three referenced Community-Based Participatory Research (CBPR), with two of these studies working with Indigenous populations and incorporating “two-eyed seeing” (Anang et al., 2019; Katapally, 2020), six used Patient and Public Involvement and/or the INVOLVE framework, five Participatory Action Research, with a further nine studies using participatory design, co-design or co-production. Some studies named specific models such as the McCain Model of Youth Engagement (Sheikhan et al., 2021) and Youth GO (Standley, 2023). References to methodologies were less specific in the remaining studies with one referencing a “stakeholder engaged approach” (Schwartz et al., 2020) and others simply describing their approach as “participatory”. Some studies combined approaches, for example co-production and PPI (Babbage et al., 2024). Nineteen studies provided a definition or at least a brief explanation of the involvement methodology they utilised.

Intervention development, design or adaptation (usually of digital interventions) was the most common stage for involvement (n = 18), often through co-design workshops. Studies also reported young people being involved in study design and planning (n = 9), recruitment and data collection (n = 10), analysis (n = 10) and dissemination (n = 8). Involvement was less common at the very start and end of a project i.e. commissioning (n = 3) and evaluation (n = 2). Eleven studies reported providing training to support young people’s involvement, but this was not clearly described or was not applicable in other studies.

## Barriers and Facilitators to Involvement of Under-Represented Young People

**Fig. 3:**
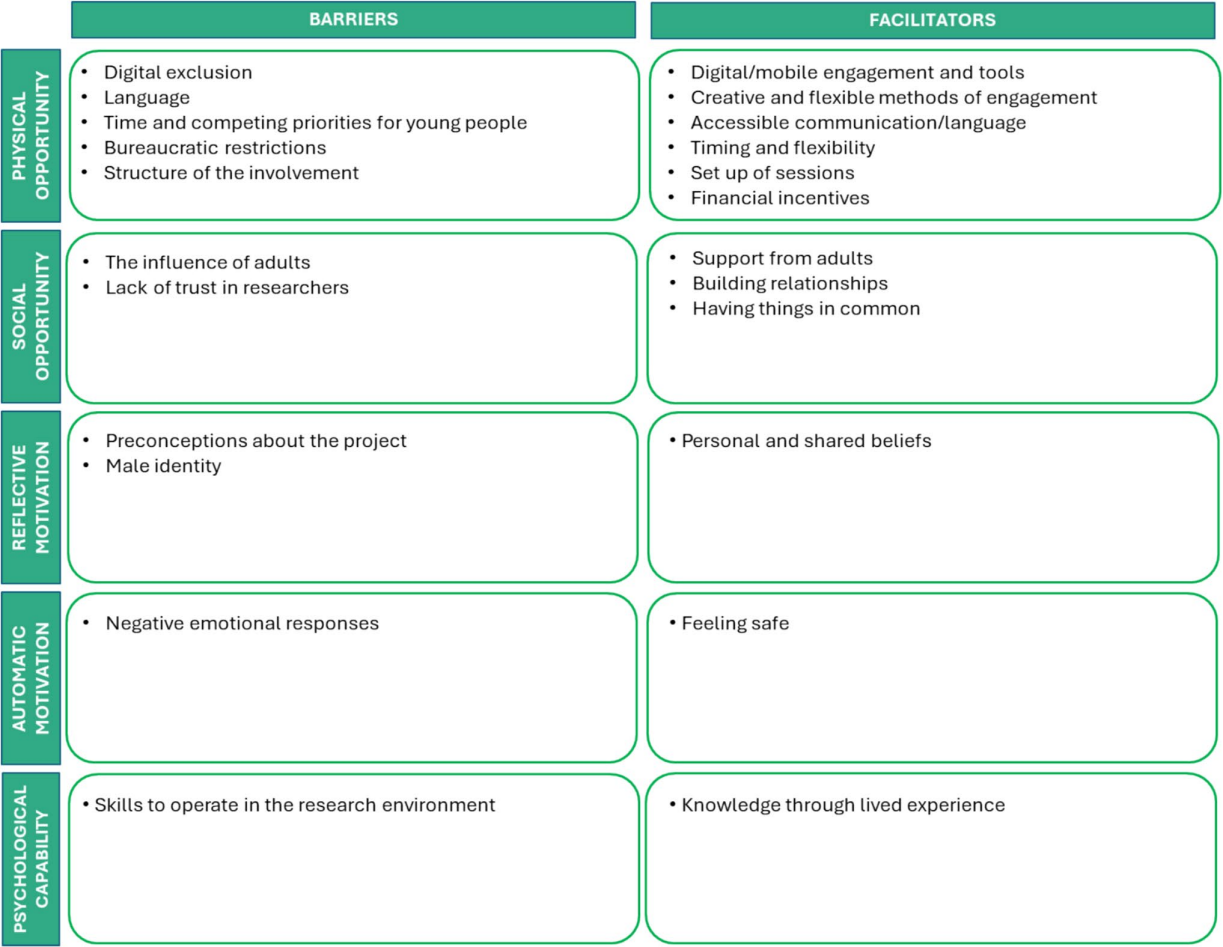
Themed barriers and facilitators mapped to the COM-B model

All but two of the included studies reported at least one barrier to under-represented young people’s involvement. Overall, 11 different barriers to involvement were reported. Five of these related to physical opportunity, two to social opportunity, two to reflective motivation and one each to automatic motivation and psychological capability. No barriers were reported relating to physical capability. Nineteen studies reported one of more facilitators. Following a similar pattern to the barriers, of the 12 facilitators, six related to physical opportunity, three to social opportunity and one to each of reflective motivation, automatic motivation and psychological capability, with no physical capability facilitators. Eighteen studies included researcher reported barriers and facilitators, two included barriers reported solely by young people (Randall, 2018; Thomson et al., 2022) and other studies reported from both perspectives. In response to RQ2, Fig. 3 illustrates these thematic barriers and facilitators coded to the corresponding COM-B domains. Tables 3 and 4 provide the barriers and facilitators, along with descriptions, which studies mention them and (to address RQ3) which demographics they related.

**Table 3:** Barriers to young people’s involvement with definitions mapped to COM-B model

COM-B area	Theme	Description	Studies	Relevant demographic groups
Physical opportunity	Digital exclusion	Where involvement activities are carried out remotely, there is varying access to digital communications and equipment to allow this, for example, lack of stable internet or the technology required. In addition, certain channels of communication were less preferred by young people e.g. post and email were seen as less convenient, and email and video conference did not allow the same degree of interaction	4 studies (Blueprint Writing Collective, 2022; Inge et al., 2024; Libon et al., 2023; Thomson et al., 2022)	Those from lower socio-economic groups/in digital poverty, refugee youth
	Language	There were a number of ways in which language influenced young people’s ability to be involved in research, these included, creating materials that were not written in accessible language, offering translation or the ability for young people to take part using their first language but also the fact that the inclusion criteria for the involvement opportunity required a particular level of English (or equivalent)	6 studies (Cheng et al., 2021; Inge et al., 2024; Povey et al., 2022; Randall, 2018; Savaglio et al., 2025; Viksveen et al., 2022)	Refugee youth, Aboriginal or Culturally and Linguistically Diverse (CALD) Young People, young people of different ethnic and cultural backgrounds
	Time and competing priorities	Some involvement projects using participatory methods (CBPR, PAR, PR) involve a significant time commitment at a time when young people have many commitments (such as studying, work, and, particularly for young people from certain communities, caring responsibilities etc.) over a prolonged period and during a stage in their life of changing circumstances such as moving from school	7 studies (Anang et al., 2019; Culbong et al., 2023; Hargrove, 2019; Katapally, 2020; Mance et al., 2010; Povey et al., 2020, 2022)	Young people from indigenous, CALD and ethnic minority backgrounds
	Bureaucratic restrictions	Researchers or young people may have wanted young people’s involvement but inclusion criteria were either restricted by the project (specifying particular ages, ethnicities etc.), by institutional hurdles such as difficulties involving young people (under 18 or 16 years of age) for ethical and consent reasons	7 studies (Babbage et al., 2024; Blueprint Writing Collective, 2022; Figueroa et al. 2025; Hargrove, 2019; Hetrick et al., 2018; Randall, 2018; Schwartz et al., 2020)	Age and ethnicity
	Structure of the involvement	The way the project had been set up could hinder engagement in terms of the types of organisations approached to recruit young people, activities, processes, lack of clear roles, expectations and structure as well as the timings or length of meetings, or not providing sufficient opportunities for young people’s involvement – either because of limiting the number of young people involved or not involving them in some aspects of the project	6 studies (Figueroa et al. 2025; Hargrove, 2019; Hetrick et al., 2018; Randall, 2018; Schwartz et al., 2020; Standley, 2023)	Ethnic minority youth, particular religious of cultural groups
Social opportunity	The influence of adults	Where groups (e.g. focus groups, workshops or meetings) included both adults and young people, young people could be discouraged or less comfortable to speak up or, on occasion, adults would speak for the young people potentially in some cases, as young people’s contributions were seen as less important. The influence of adults was mentioned in particular in terms of the cultural authority of community Elders in some studies. The inaccessible language used by adults (for example researchers) could affect engagement	7 studies (Cheng et al., 2021; Culbong et al., 2023; Dosso et al., 2024; Hargrove, 2019; Inge et al., 2024; Rocha et al., 2023; Savaglio et al., 2025)	Ethnicity/Aboriginal/Culturally and Linguistically Diverse and refugee youth, Intellectual and developmental disability, younger age groups
	Lack of trust in researchers	Lack of trust in researchers or research institutions, based on previous experience	3 studies (Anang et al., 2019; Hargrove, 2019; Mance et al., 2010)	Culturally (Inuit) and ethnically diverse youth (African American)
Psychological capability	The ability to operate in a research environment	For some young people being involved in research is a new experience and so they do not initially have the skills and knowledge needed to feel comfortable in this environment in terms of structure, mental health literature and other cognitive capabilities	3 studies (Mance et al., 2010; Stoyanov et al., 2021; Thomson et al., 2022)	Ethnic minority youth
Reflective motivation	Preconceptions about the project	Scepticism from some young people about the research they were being asked to get involved with, questioning the motives behind the initiative, the process and the longevity	2 studies (Anang et al., 2019; Hargrove, 2019)	Indigenous, ethic minority youth
	Male identity	Being a young male was a barrier in one case because young men reported feeling no need to get involved. Other specific reasons were unclear although perceptions about the environment, stigma and low mental health reasons are suggested	3 studies (Culbong et al., 2023; Randall, 2018; Stoyanov et al., 2021)	Young males
Automatic motivation	Negative emotional responses	This included a range of young people’s emotions such as discussing potentially triggering topics, worries about their contributions and not being in the mood on the day	3 studies (Hargrove, 2019; Povey et al., 2020; Thomson et al., 2022)	Ethnic minority and CALD youth

**Table 4:** Facilitators to young people’s involvement with definitions mapped to COM-B model

COM-B area	Theme	Description	Studies	Relevant demographic groups
Physical opportunity	Creative and varying methods of engagement	Workshops, “going round the table”, interactive exercises, visual research methods as well as the use of online and informal tools such as WhatsApp and other smartphone based communication	5 studies (Dosso et al., 2024; Inge et al., 2024; Katapally, 2020; Stoyanov et al., 2021; Viksveen et al., 2022)	Refugee, indigenous and IDD youth
	Accessible communication	Short documents in accessible language, use of interpreters or same language co-researchers or providing language options	6 studies (Inge et al., 2024; Pérez-Aronsson et al., 2022; Povey et al., 2020, 2022; Stoyanov et al., 2021; Viksveen et al., 2022)	4 of the studies were with refugee, migrant or indigenous youth
	Financial incentives	Providing incentives that young people value, in a timely way	4 studies (Babbage et al., 2024; Hargrove, 2019; Katapally, 2020; Liegghio, 2013)	Ethnic minority and indigenous youth and those from lower socio-economic groups
	Timing, convenience and flexibility	Scheduling involvement at opportune moments in young people’s lives and allowing flexibility in attendance, location and method	5 studies (Dosso et al., 2024; Figueroa et al. 2025; Hargrove, 2019; Inge et al., 2024; Walker et al., 2021)	Refugee, ethnic minority, Youth with long-term conditions/IDD
	Set up of sessions	Adapting the structure of involvement to suit young people e.g. young people only, different methods of interaction, flexibility in structure	3 studies (Hargrove, 2019; Inge et al., 2024; Walker et al., 2021)	Youth with long-term conditions, refugee and ethnic minority youth
Social opportunity	Building relationships	Spending time getting to know each other at the outset – both young people and researchers—on a personal level, to foster open and honest communication,	5 studies (Anang et al., 2019; Figueroa et al. 2025; Hargrove, 2019; Inge et al., 2024; Mance et al., 2010)	Indigenous, ethnic minority and refugee youth
	Support from adults	Having adults who are seen as allies, providing support and validation, giving the young people confidence and voice	3 studies (Figueroa et al. 2025; Hargrove, 2019; Rocha et al., 2023)	Latinx and ethnic minority youth
	Having things in common	Shared experiences or demographics (e.g. age, culture, mental health experiences) helped young people feel comfortable, relate to each other and be more open	4 studies (Anang et al., 2019; Figueroa et al. 2025; Hargrove, 2019; Randall, 2018)	Indigenous and ethnic minority youth
Psychological capability	Knowledge through lived experience	Being the target demographic and having that lived experience and knowledge helped to add value	1 study (Culbong et al., 2023)	Indigenous youth
Reflective motivation	Personal and shared benefits	Beliefs about how their involvement would benefit them (in terms of future career, peer support, financially etc.) as well as their community (to fill gaps, be part of the solution, addressing inequalities)	2 studies (Hargrove, 2019; Katapally, 2020)	Indigenous and ethnic minority youth
Automatic motivation	Feeling safe	Where the right environment was created (for example through group structure, accessible language, a sense of equality etc.) young people felt comfortable and safe to contribute	4 studies (Culbong et al., 2023; Hargrove, 2019; Inge et al., 2024; Randall, 2018)	Indigenous, ethnic minority and refugee youth

## Strategies Proposed to Optimise Involvement

A number of strategies were proposed across the included studies to support the involvement of under-represented young people in mental health research. Strategies tended to be generic and aimed at improving diversity overall, rather than targeting specific groups. In response to RQ4, Table 5 reports these according to the corresponding Intervention Function (Michie et al., 2014). Over half of the studies (n = 15) suggested at least one such strategy.

**Table 5:** Themed strategies suggested by studies to improve diversity and representation

Theme	Intervention Function	Examples	Studies
Communication tailored to young people’s needs	Enablement	- Provide “easy to read” materials and use terms that young people themselves use and understand to create a common language - Use different methods of presenting information such as video or visual illustrations - Adapt communication to the young people’s age, language skills etc - Making clear how a study is relevant to the lives of young people	3 studies (Inge et al., 2024; Randall, 2018; Thomson et al., 2022)
Creating supportive and safe engagement spaces	Environmental restructuring	- Creating an atmosphere of safety, respect and accountability - Provide refreshments in long meetings - Have dedicated opportunities for specific groups who may not feel comfortable in mixed groups - In mixed groups ensure there is more than just one young person - Highlight similarities between researchers and the young people involved e.g. shared mental health experience. Using young co-researchers or research ambassadors can achieve this - Offer support options and self-care strategies and tools	3 studies (Hargrove, 2019; Inge et al., 2024; Randall, 2018)
Flexible and accessible involvement methods	Environmental restructuring	- Offering flexible hours/timings and channels for involvement - Ask the young people how they want to be involved e.g. meeting times, communication channels, methods of involvement etc - Arranging updates or alternative methods of contributing for those missing meetings - Accept that timelines may need to flex to accommodate young people’s availability	5 studies (Blueprint Writing Collective, 2022; Figueroa et al. 2025; Inge et al., 2024; Savaglio et al., 2025; Walker et al., 2021)
Inclusive and diverse recruitment strategies	Environmental restructuring	- Advertising involvement opportunities beyond existing networks and channels. For example to capture those disengaged from school and work, those from ethnic or sexual minorities, younger age groups who may have less experience of services, research and other systems - Recruit specifically from within target populations - Work with young people to come up with creative/innovative recruitment strategies - Invest time to connect and work with local community groups	7 studies (Babbage et al., 2024; Blueprint Writing Collective, 2022; Cheng et al., 2021; Figueroa et al. 2025; Povey et al., 2022; Sheikhan et al., 2021; Viksveen et al., 2022)
Investing time to build trust and relationships	Enablement	- Spending time at the outset on “getting to know you” activities - Giving young people positions of responsibility and leadership to demonstrate trust - Researchers going out to young people where they are i.e. into their communities, in to the projects - Allowing sufficient time to implement the involvement, particularly for participative approaches which require significant investment of time	2 studies (Mance et al., 2010; Rocha et al., 2023)

While this review initially aimed to identify strategies used to overcome specific barriers, few studies explicitly made this link. Where this connection was clear, strategies included: supporting young people to foster group accountability in response to involvement dipping (Hargrove, 2019); providing accessible materials to overcome language challenges (Inge et al., 2024); building trust through providing young people with formal roles where mistrust was a barrier (Mance et al., 2010); recruiting outside of school settings to include those not in school (Povey et al., 2020); recruiting across age ranges to address concerns around “ageing out” (Sheikhan et al., 2021); and offering translation options to include CALD youth (Inge et al., 2024).

Of nine possible Intervention Functions, only two were reported to be used in practice: enablement and environmental restructuring.

## Quality Appraisal

The QRIPPAT appraisal shows relatively high levels of involvement reporting, especially in unpublished dissertations and theses. All studies achieved a reporting rating of six or higher out of ten on the QRIPPAT tool with the average (mean) rating being 8.6. Certain criteria, such as including the term used for involvement and describing involvement activity, were reported by all included studies. Conversely, relatively few studies described specific involvement models or evaluated impact and only one used a formal reporting tool like GRIPP2. Some studies provided only minimal discussion of barriers or facilitators, limiting the depth of analysis for these particular studies, while others gave detailed accounts of involvement processes, providing richer data. Reporting on the demographics of the young people involved was also highly variable. A table of the full QRIPPAT appraisal can be found in the supplementary materials.

## Discussion

## Research Summary

The aim of this review was to identify empirical research reporting barriers and facilitators to the involvement of under-represented young people in mental health research. From over 13,000 unique papers retrieved through the comprehensive searches, 27 papers were found to report any barriers or facilitators. However, the relatively small number of included papers reported young people’s involvement in sufficient detail to enable the identification of a number of barriers and facilitators, with some common themes, as well as a number of strategies offered to enhance the involvement of diverse young people, as reported in the findings. When consulted on the results of this review the YROG expressed surprise at the low number of studies meeting the inclusion criteria, but viewed this as a reflection of the broader lack of representation in young people’s mental health research.

## Barriers and Facilitators to Involvement

The present review has identified that barriers and facilitators were predominantly themed around opportunity-related factors (especially structural and environmental). They do so mainly through the lens of researchers with less direct reporting from under-represented young people themselves. This focus may reflect researchers having greater insight into structural and environmental influences than those relating to young people’s motivations or capabilities, particularly those of young people who have not been involved in research. Some of the most commonly reported barriers were language difficulties, time constraints, bureaucratic restrictions, involvement structures and the influence of adults. Key facilitators included accessible communication, relationship-building, creativity in engagement, timing and convenience. Many opportunity-related barriers had corresponding facilitators, providing ready-made strategies. For example, digital exclusion was countered by offering flexible methods of engagement, including both online and in-person options. This suggests researchers need not avoid digital methods, but rather use them in combination with traditional approaches. Certain groups experienced specific challenges. For example, digital exclusion affected those from lower socio-economic backgrounds, language barriers were particularly relevant for refugee and Indigenous youth, and identity and stigma were noted as distinct barriers for young men. Strategies proposed in the included studies aligned with the BCW's Intervention Functions of environmental restructuring and enablement, through flexible scheduling, inclusive recruitment, and building trust through culturally sensitive, community-based approaches.

A number of parallels with existing literature were found; a recent umbrella review (Warraitch et al., 2023) highlights the importance of inducting young people, adapting meeting formats and accommodating their personal needs and situations. The structure of involvement was highlighted as a key barrier in the present review. Similar issues are echoed in other reviews of young people’s involvement in mental health and health research more broadly, particularly with respect to adapting working practices, which requires significant time and resources (McCabe et al., 2023; Totzeck et al., 2024).

Also consistent with the wider literature (McCabe et al., 2023; Warraitch et al., 2023), language was another physical opportunity barrier, particularly for younger ages and those for whom English was not a first language, such as refugee and Indigenous youth. Strategies such as providing interpreters, simplifying documents and using visual materials help to address this challenge.

Social opportunity factors were also frequently reported. Adults’ influence can be seen as a “double-edged sword”; acting as a barrier when young people feel less able to speak freely in the presence of senior figures, but as a facilitator when adults enable their voices to be heard. The YROG, when consulted on these results agreed, believing that mixed adult/young people’s groups could be disempowering for young people, and expressed concern that some researchers can be dismissive of young people’s contributions. Power dynamics, particularly in schools, have been identified elsewhere as a challenge to involvement (Warraitch et al., 2023). Trust is therefore key but mistrust was recognised as a barrier in the present review, particularly for Indigenous and racially minoritised young people. It may be for this reason that, as illustrated in Table 2, studies involving Indigenous youth (in Australia and Canada) and racially minoritised youth in the US, employed more participatory methods such as PAR and CBPR which emphasise long-term relationships and community-led research. CBPR approaches typically position those involved as research originators rather than contributors (Cook et al., 2019). In contrast, Northern European studies (UK, Sweden, Norway) more often used PPI, co-design, or co-production, models more familiar to UK researchers. These differences may reflect national variations in priorities, research infrastructure or experience with marginalised communities (Koster et al., 2012). With over 200 involvement frameworks available in the literature, identifying the most appropriate approach is potentially daunting for new researchers (Rao et al., 2024).

Fewer barriers relating to motivation and capability were reported but these were particularly relevant to under-represented young people, such as Indigenous or racially minoritised youth. Lack of experience in research environments and negative preconceptions about research were linked to anxiety and fear, either experienced by young people or anticipated by researchers. These barriers could be mitigated when young people felt their lived experience was valued and a sense of safety was fostered. Other reviews, although not specifically focussed on under-represented groups, identified similar influences (McCabe et al., 2023; Totzeck et al., 2024; Warraitch et al., 2023).

The majority of the barriers and facilitators identified in this review mirror those reported in earlier reviews, including studies that directly report young people’s perspectives (Rao et al., 2024). However, one distinguishing finding from this review was the identification of male identity and associated mental health stigma as specific barriers to involvement. This has not been widely reported in previous reviews, although addressing male stigma has been cited as a facilitator in non-academic literature (Thomson, 2024). The present review also found that perceived similarities or shared experiences with others young people enhanced involvement, a factor that may be especially important for under-represented or marginalised young people.

## Differences in Barriers and Facilitators

A key contribution of this review lies in its group-specific analysis of barriers and facilitators, offering insight into the distinct experiences of under-represented young people. Unlike earlier reviews, this provides new insight into how involvement barriers vary across different under-represented groups**.** While this review did not uncover a wide range of previously unreported barriers or facilitators overall, this analysis suggests that important, distinct challenges do exist for under-represented young people. For example, young people with disabilities or long-term health conditions benefited from flexibility in communication and scheduling, which helped accommodate their health-related needs. Those from lower socio-economic backgrounds faced particular challenges around digital exclusion, when involvement was conducted solely online. For ethnic minority youth and those from culturally and linguistically diverse backgrounds, the importance of building trust and confidence with both the researchers and other young people is highlighted. Similarly, for immigrant youth and those communicating in a second language, accessible communication methods and feeling safe in interpersonal interactions are important and as mentioned, for young males, perceptions of mental health itself could be a barrier to involvement, while for younger adolescents, bureaucratic procedures around consent and ethics present additional challenges. Additional barriers may remain unidentified due to the limited number of studies that focus specifically on these groups or on the barriers and facilitators reported directly by young people. For example, there are very few barriers reported in the studies included in this review that relate to young people’s motivations. Motivation might be particularly influential for young people who have not been involved in research and are therefore, by definition under-represented.

## Strategies to Optimise Involvement

Table 5 summarises strategies to enhance involvement of under-represented groups, some of which overlap with, or are natural extensions of, facilitators reported in the papers. These strategies also closely align with recommendations presented in two recent guides to inclusive involvement in youth mental health research (Centring Young People in Mental Health Research—Ensuring Diversity & Inclusivity, 2023; Thomson, 2024). Notably, the present review highlights several additional strategies not featured in these resources, including the provision of support and self-care tools for young people, as well as paying careful attention to group composition to foster familiarity and a comfortable environment for young people. Strategies proposed in the papers within in this review were all categorised under the Intervention Functions of environmental restructuring or enablement. The BCW identifies these Intervention Functions as effective for tackling barriers related to opportunity (both physical and social). There were also barriers identified within the included papers for which no strategies were identified. The BCW suggests additional, evidence-based Intervention Functions which could be used to successfully overcome these barriers. For example, persuasion could be used to overcome negative perceptions of young people about researchers and the research process. A previous study in a clinical healthcare setting found that when strategies to overcome barriers were developed based on intuition rather than theory, they only matched evidence-based behaviour change strategies a small proportion of the time (Taylor et al., 2020), further underlining the value of a theory-based approach.

## Strengths and Limitations

A strength of this systematic review was the comprehensive search strategy used. The breadth of databases, grey literature and search terms ensured a broad range of involvement was captured, such as CBPR, that may not have been found in other reviews (Ali et al., 2023; Sales et al., 2021). A further significant strength was the high level of involvement of young people from the outset, both as advisors and co-researchers, actively contributing to the data collection process and decision making. In total the young people dedicated approximately sixty hours to this review, making significant personal investments of time. This level of involvement was possible due to the commitment and dedication of the young people as well as their willingness to fit the research activities into their busy schedules. The involvement required flexibility on the part of the researchers, including holding meetings and training with the young people in the evenings and weekends. The contributions of the young people were of equal quality to other authors, as demonstrated by the high levels of agreement in second reviewing.

Although the review’s search strategy was designed to be as comprehensive as possible, there is a risk that some relevant studies were not identified. Despite using a broad range of search terms, the diversity of language used to describe "involvement" in research means that studies employing less common or atypical terminology may have been missed. Similarly, the concept of "under-represented" is inherently broad and not universally defined. This review drew on existing literature to identify specific groups, recognised by researchers, as under-represented in mental health research. These categories were ethnicity, socio-economic status, first language, immigration status (immigrant, asylum seeker, refugee), disability or complex health needs, age and sex/gender. These parameters were set to ensure clarity, consistency and replicability within the scope of this review. However, it is likely that barriers for other under-represented groups were not captured. For example, when consulted on the results of this systematic review, the YROG suggested under-represented groups not covered by this review and the additional barriers to these groups that they felt existed. These barriers included travel for rural young people, girls or young women from certain cultures or religious groups not feeling able to participate in studies run by male researchers, time restrictions preventing young carers from being involved and fear of disclosure making some young people (e.g. LGBTQIA + youth) reluctant to get involved.

Additionally, limited reporting of involvement is a known issue within health and social research (Jones et al., 2021), and specifically in research with young people (Bakermans‐Kranenburg & van IJzendoorn, 2024; Sellars et al., 2021). This makes it difficult for researchers both to accurately assess who is excluded, and to build on others’ experiences (Hughes & Duffy, 2018; Jones et al., 2021). Although tools like GRIPP2 (Staniszewska et al., 2017) support transparency, ethical concerns around sharing potentially identifiable data remain, particularly when young people are co-authors. Involving young people in decisions about what to report or summarising demographics at the group level, may address these concerns.

Finally, the QRIPPAT tool was useful for assessing the reporting of involvement in the included studies. However, applying objective, evidence-based ratings to each criterion was challenging, partly due to the tool’s novelty and limited validation. This limitation is acknowledged by the tool’s authors (Rouncefield-Swales et al., 2021).

## Implications

This review highlights the need for inclusive and adaptive research practices to ensure that a diverse range of young people are meaningfully engaged in mental health research. Examples of effective good practice are provided. These include creative and flexible set up of involvement opportunities (e.g. interactive workshops, the use of visual tools and informal digital communication), accessible communication (including short, clear documents and language support), investment in relationships and timely financial incentives, which can be adopted by researchers and practitioners in the field. Strategies such as diverse recruitment approaches and creating safe spaces are proposed as potentially effective in overcoming barriers, as they alter the involvement environment and enable young people. However, inconsistent and limited reporting of involvement, particularly in terms of the demographics of those involved, hampers our understanding of what works and for whom. Wider use of reporting tools such as GRIPP2 and QRIPPAT would improve transparency and make evidence on young people’s involvement more accessible.

As suggested by the YROG, future research should include directly exploring young people’s lived experiences of barriers rather than relying predominantly on researcher report and observation, and investigating the challenges researchers face in achieving diversity. This research should include engagement with under-represented young people, in particular those with no previous research involvement, to capture their perspectives on barriers and facilitators. Moreover, given the limited reporting of specific strategies to improve involvement of particular groups, there is a need to co-produce approaches with diverse youth, explore underused Intervention Functions and publish the findings in the academic literature. Addressing these gaps will help improve equity and ensure that young people's voices are effectively integrated into shaping future mental health research.

## Conclusion

This review aimed to explore the barriers and facilitators to involving under-represented young people in mental health research, and to identify strategies to optimise involvement in this context, through application of the BCW. Opportunity-related barriers, such as accessibility issues, bureaucratic restrictions and trust in researchers were the most frequently reported barriers to involvement in mental health research for under-represented young people. Key facilitators included flexible approaches, inclusive communication, and strong relationship-building, all of which supported greater engagement. Although some capability and motivational barriers were identified, these appeared less frequently. This is likely reflecting limitations in reporting practices – most often done from the researcher’s perspectives – rather than a true absence of such barriers. To the best of our knowledge, this is the first systematic review of young people’s involvement in mental health research to apply the BCW in this way. The findings demonstrate the value of using this theoretical framework to better understand and categorise behavioural influences. An aspect of the value of using the BCW is that the findings from this review can be taken forward in future research to more systematically link barriers to evidence-based, targeted strategies for enhancing youth involvement, which this review shows represents a gap in the current literature.

## Supplementary Information

Below is the link to the electronic supplementary material.

Supplementary file1 (DOCX 61 KB)

## Data Availability

No datasets were generated or analysed during the current study.
