# Supplementary material for: Barriers and Facilitators to The Involvement of Under-Represented Children and Young People (aged 8–25) in Mental Health Research – a Systematic Review
Source: Clin Child Fam Psychol Rev. 2025 Sep 22;28(4):858–81. doi: 10.1007/s10567-025-00544-4 (PMC12660437; doi:10.1007/s10567-025-00544-4)
Supplement: Supplementary file 1 — Supplementary file1 (DOCX 61 KB) [file 10567_2025_544_MOESM1_ESM.docx]

**Article Title:** Barriers and Facilitators to The Involvement of Under-Represented Children and Young People (aged 8–25) in Mental Health Research – a Systematic Review

**Journal:** Clinical Child and Family Psychology Review

**Authors:** Rachel Perowne, Sarah Rowe, Azin Lajevardi, Luke Bingham, Ella Parry, Gabrielle Grey, Pamela Carien Thomas, Leslie Morrison Gutman

**Corresponding Author:** Leslie Morrison Gutman, UCL Centre for Behaviour Change, Division of Psychology and Language Sciences, University College London, London, UK, [l.gutman@ucl.ac.uk](mailto:l.gutman@ucl.ac.uk)

**Supplementary Material File**

1. **Final Search strategies**

CINAHL

#QueryLimiters/ExpandersLast Run ViaResultsS31S9 AND S28 AND (S29 OR S30)Expanders - Apply equivalent subjects
Search modes - Boolean/PhraseInterface - EBSCOhost Research Databases
Search Screen - Advanced Search
Database - CINAHL Plus3,552S30S16 or S17 or S18 or S19 or S20 or S21Expanders - Apply equivalent subjects
Search modes - Boolean/PhraseInterface - EBSCOhost Research Databases
Search Screen - Advanced Search
Database - CINAHL PlusDisplayS29S5 AND (S10 or S11 or S12 or S13 or S14 or S15 or S22 or S23 or S24 or S27)Expanders - Apply equivalent subjects
Search modes - Boolean/PhraseInterface - EBSCOhost Research Databases
Search Screen - Advanced Search
Database - CINAHL PlusDisplayS28research or study or studies or project* or investigat*Expanders - Apply equivalent subjects
Search modes - Boolean/PhraseInterface - EBSCOhost Research Databases
Search Screen - Advanced Search
Database - CINAHL PlusDisplayS27S25 AND S26Expanders - Apply equivalent subjects
Search modes - Boolean/PhraseInterface - EBSCOhost Research Databases
Search Screen - Advanced Search
Database - CINAHL PlusDisplayS26(involv* or orient* or collaborat* or partner* or engag*) n4 (research or trial* or method*)Expanders - Apply equivalent subjects
Search modes - Boolean/PhraseInterface - EBSCOhost Research Databases
Search Screen - Advanced Search
Database - CINAHL PlusDisplayS25patient* or public* or youth* or young* or child* or adolescen*) n2 (involv* or collaborat* or partner* or engag* or orient*)Expanders - Apply equivalent subjects
Search modes - Boolean/PhraseInterface - EBSCOhost Research Databases
Search Screen - Advanced Search
Database - CINAHL PlusDisplayS24"co-produc*" or "co produc*" or "coproduc*" or "co-design*" or "co design*" or "codesign*" or "co-creat*" or "co creat*" or "cocreat*" or "co-reserach*" or co research*" or coresearch*" or "co-investigat*" or co investigat*" or coinvestigat*"Expanders - Apply equivalent subjects
Search modes - Boolean/PhraseInterface - EBSCOhost Research Databases
Search Screen - Advanced Search
Database - CINAHL PlusDisplayS23"participatatory action research"Expanders - Apply equivalent subjects
Search modes - Boolean/PhraseInterface - EBSCOhost Research Databases
Search Screen - Advanced Search
Database - CINAHL PlusDisplayS22"patient and public involvement" or "patient and public involvement and engagement*" or "patient and public engagement*"Expanders - Apply equivalent subjects
Search modes - Boolean/PhraseInterface - EBSCOhost Research Databases
Search Screen - Advanced Search
Database - CINAHL PlusDisplayS21YPAR* or YPAG*Expanders - Apply equivalent subjects
Search modes - Boolean/PhraseInterface - EBSCOhost Research Databases
Search Screen - Advanced Search
Database - CINAHL PlusDisplayS20(youth or young or child*) n1 (advisor* or representat* or reviewer* or consult*)Expanders - Apply equivalent subjects
Search modes - Boolean/PhraseInterface - EBSCOhost Research Databases
Search Screen - Advanced Search
Database - CINAHL PlusDisplayS19("youth-adult partnership*" or "youth adult partnership*" or "child-adult partnership*" or "child adult partnership*")Expanders - Apply equivalent subjects
Search modes - Boolean/PhraseInterface - EBSCOhost Research Databases
Search Screen - Advanced Search
Database - CINAHL PlusDisplayS18(youth or "young person*" or "young people*" or child or children*) n1 ("advisory board*" or "advisory group*" or "advisory committee*")Expanders - Apply equivalent subjects
Search modes - Boolean/PhraseInterface - EBSCOhost Research Databases
Search Screen - Advanced Search
Database - CINAHL PlusDisplayS17youth n1 (involvement or participation or engagement)Expanders - Apply equivalent subjects
Search modes - Boolean/PhraseInterface - EBSCOhost Research Databases
Search Screen - Advanced Search
Database - CINAHL PlusDisplayS16"child-led research*" or "child led research*" or "youth-research*" or "youth research*" or "youth led research*" or "youth-led research*"Expanders - Apply equivalent subjects
Search modes - Boolean/PhraseInterface - EBSCOhost Research Databases
Search Screen - Advanced Search
Database - CINAHL PlusDisplayS15("user-led" or "user led" or "peer-led" or "peer led" or "consumer led" or "consumer-led" or "social action" or "social-action") n2 researchExpanders - Apply equivalent subjects
Search modes - Boolean/PhraseInterface - EBSCOhost Research Databases
Search Screen - Advanced Search
Database - CINAHL PlusDisplayS14"peer model*" or "peer-model*" or "peer research*" or "peer-research*"Expanders - Apply equivalent subjects
Search modes - Boolean/PhraseInterface - EBSCOhost Research Databases
Search Screen - Advanced Search
Database - CINAHL PlusDisplayS13"(participatory n1 (research* or model* or method* or approach* or design*)"Expanders - Apply equivalent subjects
Search modes - Boolean/PhraseInterface - EBSCOhost Research Databases
Search Screen - Advanced Search
Database - CINAHL PlusDisplayS12(MH "Action Research")Expanders - Apply equivalent subjects
Search modes - Boolean/PhraseInterface - EBSCOhost Research Databases
Search Screen - Advanced Search
Database - CINAHL PlusDisplayS11(MH "Citizen Science")Expanders - Apply equivalent subjects
Search modes - Boolean/PhraseInterface - EBSCOhost Research Databases
Search Screen - Advanced Search
Database - CINAHL PlusDisplayS10(MH "Patient Participation")Expanders - Apply equivalent subjects
Search modes - Boolean/PhraseInterface - EBSCOhost Research Databases
Search Screen - Advanced Search
Database - CINAHL PlusDisplayS9S6 OR S7 OR S8Expanders - Apply equivalent subjects
Search modes - Boolean/PhraseInterface - EBSCOhost Research Databases
Search Screen - Advanced Search
Database - CINAHL PlusDisplayS8"mental health" or "mental* ill*" or "mental disorder*" or "psychiatric disorder*" or "psychiatry" or anxiety or depression or "eating disorder*" or "anorexi* or bulimi* or suicid* or "self-injur*" or "self injur*" or "self-harm*" or "self harm*" or "panic disorder*" or "obsessive compulsive disorder*" or "post traumatic stress disorder*" or "posttraumatic stress disorder*" or post-trauatic stress disorder*"Expanders - Apply equivalent subjects
Search modes - Boolean/PhraseInterface - EBSCOhost Research Databases
Search Screen - Advanced Search
Database - CINAHL PlusDisplayS7(MH "Mental Disorders+")Expanders - Apply equivalent subjects
Search modes - Boolean/PhraseInterface - EBSCOhost Research Databases
Search Screen - Advanced Search
Database - CINAHL PlusDisplayS6(MH "Mental Health") OR (MH "Mental Health Services") OR (MH "Community Mental Health Services")Expanders - Apply equivalent subjects
Search modes - Boolean/PhraseInterface - EBSCOhost Research Databases
Search Screen - Advanced Search
Database - CINAHL PlusDisplayS5S1 OR S2 OR S3 OR S4Expanders - Apply equivalent subjects
Search modes - Boolean/PhraseInterface - EBSCOhost Research Databases
Search Screen - Advanced Search
Database - CINAHL PlusDisplayS4child or children or teen* or adolescen* or youth* or "young person*" or "young people" or "young adult*"Expanders - Apply equivalent subjects
Search modes - Boolean/PhraseInterface - EBSCOhost Research Databases
Search Screen - Advanced Search
Database - CINAHL PlusDisplayS3(MH "Young Adult")Expanders - Apply equivalent subjects
Search modes - Boolean/PhraseInterface - EBSCOhost Research Databases
Search Screen - Advanced Search
Database - CINAHL PlusDisplayS2(MH "Adolescence")Expanders - Apply equivalent subjects
Search modes - Boolean/PhraseInterface - EBSCOhost Research Databases
Search Screen - Advanced Search
Database - CINAHL PlusDisplayS1(MH "Child")Expanders - Apply equivalent subjects
Search modes - Boolean/PhraseInterface - EBSCOhost Research Databases
Search Screen - Advanced Search
Database - CINAHL PlusDisplay

**Database: Embase <1974 to 2024 March 15>**
**Search Strategy:**
**1**  child/ (2167821)
**2**  adolescent/ (1824007)
**3**  young adult/ (537926)
**4**  (child or children or adolescen* or teen* or youth* or young person* or young people* or young adult*).ti,ab,kw. (2370050)
**5**  1 or 2 or 3 or 4 (4186664)
**6**  mental health/ (222973)
**7**  mental disease/ (288095)
**8**  mental health.ti,ab,kw. (291583)
**9**  mental* ill*.ti,ab,kw. (63853)
**10**  mental disorder*.ti,ab,kw. (68171)
**11**  (psychiatric disorder* or psychiatry or anxiety or depression or self-harm or self harm or self- injur* or self injur* or suicid* or eating disorder* or anorexi* or bulimi* or panic disorder* or obsessive compulsive disorder* or post traumatic stress disorder* or posttraumatic stress disorder* or post-traumatic stress disorder*).ti,ab,kw. (1136315)
**12**  6 or 7 or 8 or 9 or 10 or 11 (1539821)
**13**  participatory research/ (7571)
**14**  patient participation/ (37063)
**15**  (Participatory adj (research* or method* or design* or approach* or model*)).ti,ab,kw. (9390)
**16**  (peer-model* or peer model* or peer-research* or peer research*).ti,ab,kw. (630)
**17**  ((user-led or user led or peer-led or peer led or consumer led or consumer-led or social-action or social action) adj2 research*).ti,ab,kw. (101)
**18**  (child-led research* or child led research* or youth-research* or youth research* or youth-led research* or youth led research*).ti,ab,kw. (251)
**19**  (youth participation or youth involvement or youth engagement).ti,ab,kw. (823)
**20**  ((child or children* or youth* or young person* or young people*) adj (advisory board* or advisory group* or advisory committee*)).ti,ab,kw. (204)
**21**  (youth-adult partnership* or youth adult partnership* or child-adult partnership* or child adult partnership*).ti,ab,kw. (43)
**22**  ((youth or young or child*) adj (advisor* or representat* or reviewer* or consult*)).ti,ab,kw. (1144)
**23**  (YPAR* or YPAG*).ti,ab,kw. (113)
**24**  ((child or children* or youth* or young people* or young person*) adj2 advisory group).ti,ab,kw. (125)
**25**  ((patient and public involvement) or (patient and public engagement) or (patient and public involvement and engagement)).ti,ab,kw. (2195)
**26**  "participatory action research*".ti,ab,kw. (2466)
**27**  (co-produc* or co produc* or coproduc* or co-design* or co design* or codesign* or co-research* or co research* or coresearch* or co-creat* or co-creat* or cocreat* or co-investigat* or co investigat* or coinvestigat*).ti,ab,kw. (18380)
**28**  ((patient* or public* or youth* or young* or child* or adolescen*) adj2 (involv* or orient* or collaborat* or partner* or engag*)).ti,ab,kw. (172080)
**29**  ((involv* or orient* or collaborat* or partner* or engag*) adj4 (research or trial* or method*)).ti,ab,kw. (189388)
**30**  28 and 29 (22182)
**31**  13 or 14 or 15 or 16 or 17 or 25 or 26 or 27 or 30 (90021)
**32**  18 or 19 or 20 or 21 or 22 or 23 or 24 (2313)
**33**  5 and 31 (16692)
**34**  33 or 30 or 32 (35287)
**35**  (research or study or studies or project* or investigat*).ti,ab,kw. (19701802)
**36**  34 and 12 and 35 (3921)
**37**  limit 36 to english language (3799)

**Database: Ovid MEDLINE(R) ALL <1946 to March 15, 2024>**
**Search Strategy:**
**1**  Child/ (1953898)
**2**  exp Adolescent/ (2238145)
**3**  exp Young Adult/ (1023028)
**4**  (child or children or adolescen* or teen* or young people* or young person* or youth* or young adult*).ti,ab,kw. (1874026)
**5**  1 or 2 or 3 or 4 (4291399)
**6**  exp Mental Health/ (66158)
**7**  mental disorders/ or anxiety disorders/ or "feeding and eating disorders"/ or mood disorders/ or personality disorders/ or "schizophrenia spectrum and other psychotic disorders"/ or "trauma and stressor related disorders"/ (268687)
**8**  (mental health or mental disorder* or mental* ill* or psychiatric disorder* or psychiatry or anxiety or depression or suicid* or self-harm or self harm or eating disorder* or anorexi* or bulimi* or self-injur* or self injur* or panic disorder* or obsessive compulsive disorder* or post traumatic stress disorder* or posttraumatic stress disorder* or post-traumatic stress disorder*).ti,ab,kw. (1030348)
**9**  6 or 7 or 8 (1151366)
**10**  exp community-based participatory research/ or exp citizen science/ (6265)
**11**  community participation/ or exp patient participation/ (47982)
**12**  ((participatory or participative) adj (research* or method* or approach* or design* or model*)).ti,ab,kw. (8689)
**13**  (peer model* or peer-model* or peer research* or peer-research*).ti,ab,kw. (520)
**14**  ((user-led or user led or peer-led or peer led or consumer-led or consumer led or social-action or social action) adj2 research*).ti,ab,kw. (76)
**15**  (child-led research* or child led research* or youth-research* or youth research* or youth-led research* or youth led research*).ti,ab,kw. (187)
**16**  (youth adj (involvement or participation or engagement)).ti,ab,kw. (708)
**17**  ((youth* or young person* or young people* or child or children*) adj (advisory board* or advisory group* or advisory committee*)).ti,ab,kw. (117)
**18**  (youth-adult partnership* or youth adult partnership* or child-adult partnership* or child adult partnership*).ti,ab,kw. (39)
**19**  ((youth or young or child*) adj (advisor* or representat* or reviewer* or consult*)).ti,ab,kw. (823)
**20**  (YPAR* or YPAG*).ti,ab,kw. (82)
**21**  ((child or children* or young person* or young people* or youth*) adj2 advisory group).ti,ab,kw. (71)
**22**  ("patient and public involvement" or "patient and public engagement" or "patient and public involvement and engagement").ti,ab,kw. (1831)
**23**  "participatory action research*".ti,ab,kw. (2124)
**24**  (co-produc* or co produc* or coproduc* or co-design* or co design* or codesign* or co-creat* or co creat* or cocreat* or co-research* or co research* or coresearch* or co-investigat* or co investigat* or coinvestigat*).ti,ab,kw. (15452)
**25**  ((patient* or public* or youth* or young* or child* or adolescen*) adj2 (involv* or orient* or collaborat* or partner* or engag*)).ti,ab,kw. (119266)
**26**  ((involv* or orient* or collaborat* or partner* or engag*) adj4 (research or trial* or method*)).ti,ab,kw. (129407)
**27**  25 and 26 (14485)
**28**  10 or 11 or 12 or 13 or 14 or 22 or 23 or 24 (75959)
**29**  15 or 16 or 17 or 18 or 19 or 20 or 21 (1796)
**30**  5 and 28 (13106)
**31**  27 or 29 or 30 (28478)
**32**  (research or study or studies or project* or investigat*).ti,ab,kw. (15497973)
**33**  9 and 31 and 32 (2826)
**34**  limit 33 to english language (2746)

**Database: APA PsycInfo <1806 to March Week 3 2024>**
**Search Strategy:**
**1**  (child or children).ab,id,ti. (711173)
**2**  "adolescen*".ab,id,ti. (302124)
**3**  "young adult*".ab,id,ti. (64385)
**4**  "teen*".ab,id,ti. (25991)
**5**  (young person* or young people*).ab,id,ti. (40940)
**6**  "youth*".ab,id,ti. (126951)
**7**  1 or 2 or 3 or 4 or 5 or 6 (1003657)
**8**  exp Mental Health Services/ or exp Youth Mental Health/ or exp Mental Disorders/ or exp Mental Health/ or exp Mental Health Programs/ (1202292)
**9**  mental health.ab,id,ti. (252291)
**10**  mental disorder*.ab,id,ti. (71147)
**11**  (psychiatry or psychiatric disorder*).ab,id,ti. (118580)
**12**  mental* ill*.ab,id,ti. (62613)
**13**  (anxiety or depression or suicid* or self-harm or self harm or eating disorder* or anorexi* or bulimi* or self-injur* or self injur* or panic disorder* or obsessive compulsive disorder* or post traumatic stress disorder* or posttraumatic stress disorder* or post-traumatic stress disorder*).ab,id,ti. (586008)
**14**  8 or 9 or 10 or 11 or 12 or 13 (1524976)
**15**  exp Action Research/ (4459)
**16**  exp Community Involvement/ (7503)
**17**  (participatory adj (research* or method* or approach* or design* or model*)).ab,id,ti. (6392)
**18**  (peer model* or peer-model* or peer research* or peer-research*).ab,id,ti. (787)
**19**  ((user-led or user led or peer-led or peer led or consumer-led or consumer led or social-action or social action) adj2 research).ab,id,ti. (179)
**20**  (child-led research* or child led research* or youth-research* or youth research* or youth-led research* or youth led research*).ab,id,ti. (381)
**21**  (youth adj (involvement or participation or engagement)).ab,id,ti. (1069)
**22**  ((youth* or young person* or young people* or child or children*) adj (advisory board* or advisory group* or advisory committee*)).ab,id,ti. (63)
**23**  (youth-adult partnership* or youth adult partnership* or child-adult partnership* or child adult partnership*).ab,id,ti. (98)
**24**  ((youth or young or child) adj (advisor* or representat* or reviewer* or consult*)).ab,id,ti. (234)
**25**  (YPAR* or YPAG*).ab,id,ti. (155)
**26**  ((child or children* or young people* or young person* or youth*) adj2 advisory group).ab,id,ti. (31)
**27**  ("patient and public involvement" or "patient and public involvement and engagement" or "patient and public engagement").ab,id,ti. (508)
**28**  "participatory action research*".ab,id,ti. (2862)
**29**  (co-produc* or co produc* or coproduc* or co-design* or co design* or codesign* or co-creat* or co creat* or cocreat* or co-research* or co research* or coresearch* or co-investigat* or co investigat* or coinvestigat*).ab,id,ti. (8945)
**30**  ((patient* or public* or youth* or young* or child* or adolescen*) adj2 (involv* or orient* or collaborat* or partner* or engag*)).ab,id,ti. (52916)
**31**  ((involv* or orient* or collaborat* or partner* or engag*) adj4 (research or trial* or method*)).ab,id,ti. (57241)
**32**  30 and 31 (4434)
**33**  7 and (15 or 16 or 17 or 18 or 19 or 27 or 28 or 29) (6287)
**34**  20 or 21 or 22 or 23 or 24 or 25 or 26 (1857)
**35**  32 or 33 or 34 (11750)
**36**  14 and 35 (2897)
**37**  (research or study or studies or project* or investigat*).ab,id,ti. (3327857)
**38**  36 and 37 (2544)
**39**  limit 38 to english language (2352)

**Web of Science Search**

**(#19 AND #20 AND #21) AND (LA==("ENGLISH"))**

[4,030](https://www-webofscience-com.libproxy.ucl.ac.uk/wos/woscc/summary/6149b717-1fb9-49fb-94b5-5de88c46e563-d61139ac/relevance/1)

Add to query

linkeditnotifications

21

**TS=(research or study or studies and project* or investigat*)**

[30,909,067](https://www-webofscience-com.libproxy.ucl.ac.uk/wos/woscc/summary/5c19bf18-135d-4ba4-831c-63d93be650ca-d3b8e2ec/relevance/1)

Add to query

linkeditnotifications

20

**TS=("mental health" or "mental disorder*" or "mental* ill*" or "psychiatric disorder*" or "psychiatry" or "anxiety" or "depression" or "eating disorder*" or "anorexi*" or "bulimi*" or "suicid*" or "self-injur*" or "self injur*" or "self-harm*" or "self harm*" or "panic disorder*" or "obsessive compulsive disorder*" or "post traumatic stress disorder*" or "posttraumatic stress disorder*" or "post-traumatic stress disorder*")**

[1,486,819](https://www-webofscience-com.libproxy.ucl.ac.uk/wos/woscc/summary/61023057-8fe3-4a4a-ab48-6a157cfbf9c1-d3b90be7/relevance/1)

Add to query

linkeditnotifications

19

**#7 OR #15 OR #18**

[42,609](https://www-webofscience-com.libproxy.ucl.ac.uk/wos/woscc/summary/cced1353-6b53-4f77-8a4f-c29d00c18ef8-d051e356/relevance/1)

Add to query

linkeditnotifications

18

**#16 AND #17**

[25,676](https://www-webofscience-com.libproxy.ucl.ac.uk/wos/woscc/summary/2d8f2805-e87b-4004-ae0f-56b3f9a9c676-d051e325/relevance/1)

Add to query

linkeditnotifications

17

**TS=((involv* or orient* or collaborat* or partner* or engag*) NEAR/4 (research or trial* or method*))**

[324,963](https://www-webofscience-com.libproxy.ucl.ac.uk/wos/woscc/summary/f3a39768-6279-47f0-91ee-6a516f2e6ef1-d051e2e0/relevance/1)

Add to query

linkeditnotifications

16

**TS=((patient* or public* or youth* or young* or child* or adolescen*) NEAR/2 (involv* or orient* or collaborat* or partner* or engag*))**

[218,738](https://www-webofscience-com.libproxy.ucl.ac.uk/wos/woscc/summary/eb32201b-cc60-4fd2-9fbe-cff6716d460e-d051db35/relevance/1)

Add to query

linkeditnotifications

15

**#8 AND (#9 OR #10 OR #11 OR #12 OR #13 OR #14)**

[10,261](https://www-webofscience-com.libproxy.ucl.ac.uk/wos/woscc/summary/b3c67d68-4a2e-4b1c-8e61-5946e70f04c4-d051cd34/relevance/1)

Add to query

linkeditnotifications

14

**TS=("co-produc*" or "co produc*" or "coproduc*" or "co-design*" or "co design*" or "codesign*" or "co-creat*" or "co creat*" or "cocreat*" or "co-research*" or "co research*" or "coresearch*" or "co-investigat*" or "co investigat*" or "coinvestigat*")**

[68,200](https://www-webofscience-com.libproxy.ucl.ac.uk/wos/woscc/summary/7b22c796-251a-44c7-a67b-bbaa015c3cd4-cf5150c6/relevance/1)

Add to query

linkeditnotifications

13

**TS=("patient and public involvement" or "patient and public engagement" or "patient and public involvement and engagement")**

[1,575](https://www-webofscience-com.libproxy.ucl.ac.uk/wos/woscc/summary/aa7712de-4544-4906-a86b-955fe5705146-d051ccff/relevance/1)

Add to query

linkeditnotifications

12

**TS=("participatory action research")**

[6,990](https://www-webofscience-com.libproxy.ucl.ac.uk/wos/woscc/summary/76a5fa46-0189-4522-8a48-1fff3786df8b-c9769f42/relevance/1)

Add to query

linkeditnotifications

11

**TS=("participatory research*" or "participatory model*" or "participatory method*" or "participatory design*" or "participatory approach*")**

[26,538](https://www-webofscience-com.libproxy.ucl.ac.uk/wos/woscc/summary/e15bd9cd-767a-4456-b32e-552313d9b6c6-cf513d4e/relevance/1)

Add to query

linkeditnotifications

10

**TS=(("user led" or "user-led" or "peer led" or "peer-led" or "consumer-led" or "consumer led" or "social action" or "social-action") NEAR/2 (research))**

[262](https://www-webofscience-com.libproxy.ucl.ac.uk/wos/woscc/summary/4d407021-9db1-4129-aa49-a91bbe9c58b0-cb32ca37/relevance/1)

Add to query

linkeditnotifications

9

**TS=("peer model*" or "peer-model*" or "peer research*" or "peer-research*")**

[1,281](https://www-webofscience-com.libproxy.ucl.ac.uk/wos/woscc/summary/d9a7d025-3563-43cd-a6d0-3ead4e2ad2b5-cf513765/relevance/1)

Add to query

linkeditnotifications

8

**TS=("young person*" or "young people" or child or children or teen* or youth* or "young adult*" or adolescen*)**

[2,823,889](https://www-webofscience-com.libproxy.ucl.ac.uk/wos/woscc/summary/22304cdf-7064-4d69-bd49-a86fa579dd5e-cf51338d/relevance/1)

Add to query

linkeditnotifications

7

**#1 OR #2 OR #3 OR #4 OR #5 OR #6**

[8,330](https://www-webofscience-com.libproxy.ucl.ac.uk/wos/woscc/summary/49e5cf85-75b2-427b-a430-1f693fda4e4e-d051b164/relevance/1)

Add to query

linkeditnotifications

6

**TS=(YPAR* or YPAG*)**

[268](https://www-webofscience-com.libproxy.ucl.ac.uk/wos/woscc/summary/ba33c3ad-8dd2-4f2c-b755-3941aa333915-ca327093/relevance/1)

Add to query

linkeditnotifications

5

**TS=((Youth or young or child*) NEAR/1 (advisor* or consult* or representat* or reviewer))**

[5,173](https://www-webofscience-com.libproxy.ucl.ac.uk/wos/woscc/summary/6623c6ee-1386-424c-9737-cb49120d6cbe-d051a66e/relevance/1)

Add to query

linkeditnotifications

4

**TS=("youth-adult partnership*" or "youth adult partnership*" or "child-adult partnership*" or "child adult partnership*")**

[145](https://www-webofscience-com.libproxy.ucl.ac.uk/wos/woscc/summary/151392ed-3bda-4c7a-9914-7f34eb3e9282-cf512aa3/relevance/1)

Add to query

linkeditnotifications

3

**TS=(("young person*" or "young people*" or child or children* or teen* or youth or "young adult*") NEAR/1 ("advisory board*" or "advisory group*" or "advisory committee*"))**

[211](https://www-webofscience-com.libproxy.ucl.ac.uk/wos/woscc/summary/765388c1-729e-4e30-b2ed-f9f76dbc4c10-d0516eeb/relevance/1)

Add to query

linkeditnotifications

2

**TS=("youth involvement" or "youth engagement" or "youth participation")**

[2,273](https://www-webofscience-com.libproxy.ucl.ac.uk/wos/woscc/summary/75b82c27-7fc9-43fd-8125-5205fa5a21f9-cf512643/relevance/1)

Add to query

linkeditnotifications

1

**TS=("child-led research*" or "child led research*" or "youth research*" or "youth-research*" or "youth led research*" or "youth-led research*")**

1. **GRIPP2 Reporting Checklist - long form**

| **Section and topic** | **Item** | **Reported in Section number (or page number where relevant)** |
| --- | --- | --- |
| **Section 1: Abstract of paper** |  |  |
| 1a: Aim | Report the aim of the study | Page 1 |
| 1b: Methods | Describe the methods used by which patients and the public were involved | Page 1 |
| 1c: Results | Report the impacts and outcomes of PPI in the study | Page 2 |
| 1d: Conclusions | Summarise the main conclusions of the study | Page 1 & 2 |
| 1e: Keywords | Include PPI, ‘patient and public involvement’ or alternative terms as keywords | Page 2 |
| **Section 2: Background to paper** |  |  |
| 2a: Definition | Report the definition of PPI used in the study and how it links to comparable studies | Section 1 (para 2) & Section 2.1 (para 3) |
| 2b: Theoretical underpinnings | Report the theoretical rationale and any theoretical influences relating to PPI in the study | Section 1 (para 2) |
| 2c: Concepts and theory development | Report any conceptual or theoretical models, or influences, used on the study | Section 1 (para 6) |
| **Section 3: Aims of paper** |  |  |
| 3. Aim | Report the aim of the study | Section 1 (para 7) |
| **Section 4: Methods of paper** |  |  |
| 4a: Design | Provide a clear description of methods by which patients and the public were involved Provide a description of patients, carers, and the public | Section 2.6 |
| 4b: People involved | Provide a description of patients, carers, and the public involved with the PPI activity in the study | Section 2.6 |
| 4c: Stages of involvement | Report on how PPI is used at different stages of the study | Section 2.1 (para 3), Section 2.2 (paras 1 and 4), Section 2.3 |
| 4d: Level or nature of involvement | Report the level of nature of PPI used at various stages of the study | Section 2.1 (para 3), Section 2.2 (paras 1 and 4), Section 2.3 |
| **Section 5: Capture of measurement of PPI impact** |  |  |
| 5a: Qualitative evidence of impact | If applicable, report the methods used to qualitatively explore the impact of PPI in the study | To be reported elsewhere |
| 5b: Quantitative evidence of impact | If applicable, report the methods used to quantitatively measure or assess the impact of PPI | To be reported elsewhere |
| 5c: Robustness of measure | If applicable, report the rigour of the method used to capture or measure the impact of PPI | To be reported elsewhere |
| **Section 6: Economic assessment** |  |  |
| 6: Economic assessment | If applicable report the method used for an economic assessment of PPI | NA |
| **Section 7: Study results** |  |  |
| 7a: Outcome of PPI | Report the results of PPI in the study, including both positive and negative outcomes | Section 3.1 |
| 7b: Impacts of PPI | Report the positive and negative impacts that PPI has had on the research, the individuals involved (including patients and researchers) and the wider impacts | Section 3.3.7 |
| 7c: Context of PPI | Report the influence of any contextual factors that enabled or hindered the process or impact of PPI | To be reported elsewhere |
| 7d: Process of PPI | Report the influence of any process factors, that enabled or hindered the impact of PPI | To be reported elsewhere |
| 7ei: Theory development | Report any conceptual or theoretical development in PPI that have emerged | NA |
| 7eii: Theory development | Report evaluation of theoretical models, if any | NA |
| 7f: Measurement | If applicable, report all aspects of instrument development and testing (eg validity, reliability, feasibility, acceptability, responsiveness, interpretability, appropriateness, precision) | NA |
| 7g: Economic assessment | Report any information on the costs or benefit of PPI | Section 2.6 (para 2) |
| **Section 8: Discussion and conclusions** |  |  |
| 8a: Outcomes | Comment on how PPI influenced the study overall. Describe positive and negative effects | Section 4.5 (para 4). To be reported in greater detail elsewhere. |
| 8b: Impacts | Comment on the different impacts of PPI identified in this study and how they contribute to new knowledge | Section 4.5 (para 4). To be reported in greater detail elsewhere. |
| 8c: Definition | Comment on the definition of PPI used (reported in the Background section) and whether or not you would suggest any changes | Section 4.1 (para 2) |
| 8d: Theoretical underpinnings | Comment on any way your study adds to the theoretical development of PPI | Section 4.1 (para 2) |
| 8e: Context | Comment on how context factors influenced PPI in the study | Section 4.5 (para 4). To be reported in greater detail elsewhere. |
| 8f: Process | Comment on how process factors influence PPI in the study | Section 4.5 (para 4). To be reported in greater detail elsewhere. |
| 8g: Measurement and capture of PPI impact | If applicable, comment on how well PPI impact was evaluated or measured in the study | NA |
| 8h: Economic assessment | If applicable, discuss any aspect of the economic cost or benefit of PPI, particularly any suggestions for future economic modelling | NA |
| 8i: Reflections/critical perspective | Comment critically on the study, reflecting on the things that went well and those that did not, so that others can learn from this study | Section 4.5 (para 4). To be reported in greater detail elsewhere. |

1. **Study descriptions**

| **First Author, Year** | **Title** | **Country** | **Research Aim** | **Design** | **Setting** | **Mental health focus** |
| --- | --- | --- | --- | --- | --- | --- |
| Anang 2019 | Building on strengths in Naujaat: the process of engaging Inuit youth in suicide prevention | Canada | To establish a partnership with a group of young people in the community and a community Elder to develop a set of goals and directions to enhance youth resiliency | Qualitative focus groups | Naujaat Community | Suicide |
| Babbage 2024 | Cultivating participatory processes in self‐harm app development: A case‐study and working methodology | UK | To share the approach and activities we have developed to deliver PPI and coproduction with young people with lived experience in the participatory (Planning and Discovery, Design and Development) phases of the CaTS‐App development. | Case study | Clinical community based mental health setting | Self-harm |
| Blueprint 2022 | A Blueprint for Involvement: Reflections of lived experience co-researchers and academic researchers on working collaboratively | UK | To provide a reflective account of lived experience co-researchers’ and academic researchers’ expectations and experiences of involvement | Collaborative reflexive approach with thematic analysis | Services for children and young people with common mental health problems | General mental health |
| Cheng 2021 | Recommendations for Designing Health Information Technologies for Mental Health Drawn From Self-Determination Theory and Co-design With Culturally Diverse Populations: Template Analysis | Australia | To identify how best to improve a mental health and well-being health information technology for culturally diverse Australians in nonurban areas. | Participatory design - co-design workshops with thematic analysis | Primary youth mental health services | General mental health |
| Culbong 2023 | “Ngany Kamam, I Speak Truly”: First-Person Accounts of Aboriginal Youth Voices in Mental Health Service Reform | Australia | To explore the impact of Aboriginal young people’s voices on mental health service reform, drawing on learnings from The Building Bridges Project | Aboriginal Participatory Action Research | Community mental health services, Whadjuk Nyoongar boodjam community | General mental health |
| Dosso 2024 | The League: A person‐centred approach to the development of social robotics for paediatric anxiety | Canada | To describe engagement with experts with lived experiences of paediatric anxiety in a social robotics research programme. | Co-creation | Youth mental health support organisation | Anxiety |
| Figueroa 2025 | Skepticism and Excitement when Co-designing Just-In-Time Mental Health Apps with Minoritized Youth | Nether-lands | To understand the wishes, needs and concerns regarding mental health apps of adolescents with a racial and ethnic minority background and living in lower income neighborhoods in the Netherlands. | Participatory research | Community centres | General mental health |
| Hargrove 2019 | A Process for Change: A Grounded Theory Investigation of Participatory Action Research as a Means for Countering Mental Illness Stigma Experienced by Transition-Aged Black Youth | US | To assess whether engaging in the process of participatory action research could effectively function as a self-stigma intervention among Black transition age youth. | Qualitative grounded theory interviews | Student body at the University of Massachusetts Boston | Mental health stigma |
| Hetrick 2018 | Youth Codesign of a Mobile Phone App to Facilitate Self-Monitoring and Management of Mood Symptoms in Young People With Major Depression, Suicidal Ideation, and Self-Harm | Australia | To co-design a self-monitoring  tool delivered via an app for young people receiving face-to-face clinical management of major depression. | Participatory design and studio design following human-centred design principles | Youth mood clinic, secondary and tertiary mental health services | Suicide, depression and self-harm |
| Inge 2024 | Ameliorating epistemic injustice in practice: Communication strategies in a research project with refugee youth coresearchers | Sweden | To evaluate the communication strategies adopted throughout a research project with refugee youth coresearchers and identify which communication strategies enabled refugee youth involvement in the evaluated project and how the communication strategies contributed to enabling involvement | Multi method qualitative approach to evaluation of communication strategies | Support groups for refugee children and youth with symptoms of posttraumatic stress | Post traumatic Stress |
| Katapally 2020 | Smart Indigenous Youth: The Smart Platform Policy Solution for Systems Integration to Address Indigenous Youth Mental Health | Canada | To improve Indigenous youth mental health by embedding a culturally appropriate digital health initiative into school curricula in rural and remote Indigenous communities in Canada | Participatory Action Research, Two Eyed Seeing, CBPR, citizen science and systems science | Schools | General mental health |
| Libon 2023 | Youth Perspectives on Barriers and Opportunities for the Development of a Peer Support Model to Promote Mental Health and Prevent Suicide | Canada | To elicit and document the perspectives of youth (ages 15–24) on the development of a peer support model for mental health promotion and suicide prevention for youth in small communities in western Canada, and identify barriers and opportunities for improving suicide prevention programming for youth | Qualitative descriptive investigation, with co-design | Community based organisations in small rural communities in Western Canada | Mental health promotion and suicide |
| Liegghio 2013 | The Stigma of Mental Illness: Learning From the Situated Knowledge of Psychiatrized Youth, Caregivers, and Young Siblings | Canada | To examine the effects of self and family stigma as individual and intersecting experiences on the subjectivities and mental health experiences of youth diagnosed with mental health issues, and on their caregivers, and siblings. | Qualitative design using Participatory Action Research | Community-based children’s mental health agency | Mental health stigma |
| Mance 2010 | Utilizing Community-Based Participatory Research to Adapt a Mental Health Intervention for African American Emerging Adults | US | To adapt a mental health intervention for adolescents and young adults ages 16 to 24 disconnected from school and work who are enrolled in an employment training program, using CBPR | CBPR | Community development organization, in East Baltimore | Depression |
| Perez-Aronsson 2022 | Adaptation of the trauma group intervention ‘Teaching Recovery Techniques’ for online delivery: A participatory design and usability study | Sweden | To adapt an in-person group intervention (Teaching Recovery Techniques) for online delivery and investigate the usability of the new intervention format. | Participatory workshops and consultation, usability testing | Children's rights organisation | PTSD |
| Povey 2022 | Determining Priorities in the Aboriginal and Islander Mental Health Initiative for Youth App Second Phase Participatory Design Project: Qualitative Study and Narrative Literature Review | Australia | To iteratively enhance an Aboriginal and Islander mental health app | Participatory design | Schools and residential drug rehabilitation facility | General mental health |
| Povey 2020 | Drafting the Aboriginal and Islander Mental Health Initiative for Youth (AIMhi-Y) App: Results of a formative mixed methods study | Australia | To explore the lived experience of mental health and wellbeing with Aboriginal and Torres Strait Islander youth in three Top End Northern Territory settings, examine the characteristics of e-mental health resources that render them acceptable and appropriate for Aboriginal and Torres Strait Islander youth and draft a culturally responsive e-mental health resource in collaboration with Aboriginal and Torres Strait Islander youth participants. | Mixed methods with participatory design | Schools and residential drug rehabilitation facility | General mental health |
| Randall 2018 | I want to do something positive with my experiences: The Youth Involvement in Mental Health Research project | Australia | To examine the characteristics, motivations and experiences of the young  people involved in youth mental health research | A multi-method approach, involving five interconnected studies. Three qualitative studies using focus groups, interviews and analysis of existing data; two quantitative studies using a longitudinal data and an online cross-sectional survey. | An Australian youth mental health and technology research  collaboration | General mental health |
| Rocha 2023 | Using Youth-Led Participatory Action Research to Advance the Mental Health Needs of Latinx Youth During COVID-19 | US | To showcase the potential of youth participatory action research as a tool for social justice and equity work in schools, answered the research question 'how well are youth doing the COVID-19 crisis? | Youth-led Participatory Action Research, nonexperimental transformational, quantitative online survey design | Schools | General mental health |
| Savaglio 2025 | Using Intervention Mapping to co-design a psychosocial service with youth experiencing mental illness | Australia | to describe the use of IM methodology to develop a community-based psychosocial service to support young people (aged 10–25 years) experiencing mental illness. | Intervention Mapping | Community service organisation | General mental illness |
| Schwartz 2020 | Stakeholder-driven approach to developing a peer-mentoring intervention for young adults with intellectual/developmental disabilities and co-occurring mental health conditions | US | To identify the prioritized outcome that a peer-mentoring intervention for young adults with intellectual/developmental disabilities and co-occurring mental health conditions (IDD-MH) should address and to identify the features and content considerations for an effective and feasible peer-mentoring intervention for young adults with IDD-MH. | Qualitative stakeholder-engaged approach using focus groups and interviews | High school students | General mental health |
| Sheikhan 2021 | ‘It reshaped how I will do research’: A qualitative exploration of team members’ experiences with youth and family engagement in a randomized controlled trial | Canada | to explore the project team's experience of youth and family engagement in the design and development of the YouthCan IMPACT randomized controlled trial and clinical service  pathway design. | Secondary study of qualitative interview data from evaluation of an RCT | Child, youth and family mental health centre | General mental health |
| Standley 2023 | “Just Somebody to Listen”: Identity, School Climate, and Mental Health Among High School Students | US | to elevate the voices of minoritized youth and leverage the novel integration of theoretical frameworks to better understand young people’s experiences of marginalization and suicidality and what they would like to see change in their schools. 1. to better understand how the intersections of identities (e.g., race and ethnicity, sexual identity, and gender identity) impact young people’s experiences of mental health suicidality. 2. to understand what youth feel they need changed in their schools to  feel more supported and affirmed | Qualitative study using a participatory approach (Youth GO) | School | Suicide |
| Stoyanov 2021 | Development of the Niggle App for Supporting Young People on Their Dynamic Journey to Well-being: Co-design and Qualitative Research Study | Australia | To explore how young Australians conceptualize and construct recovery journeys from feeling unwell to being well in order to inform the conceptual design of a youth-led information-, resource-, and support-focused mHealth intervention. | Participatory design workshops | Support service for young people | General mental health |
| Thomson 2022 | The Youth Patient and Public Involvement Café—A youth‐led model for meaningful involvement with children and young people | UK | To explore how a diverse range of CYP could become interested and engaged in the research process. 2. To develop a model of meaningful engagement of CYP in research. 3. To codevelop a programme of meaningful research ‘with’ CYP, rather than ‘for’ them a. To work with young people to establish key priorities for research. b. To generate new youth‐focused research and opportunities, to further innovate the field of CYP mental health. | Discussion forums with qualitative thematic analysis, co-development | NHS foundation Trust | General mental health |
| Viksveen 2022 | Involvement of adolescent representatives and coresearchers in mental health research: Experiences from a research project | Norway | To present experiences and reflections based on the involvement of adolescents in mental health research, to describe the collaborative relationship between researchers and coresearchers, including the values that underpin their collaboration. | Autoethnography | Upper secondary school and mental health services | General mental health |
| Walker 2021 | No evidence synthesis about me without me: Involving young people in the conduct and dissemination of a complex evidence synthesis | UK | To describe and reflect on the methods and influence of involvement of young people with lived experience within a complex evidence synthesis. | Linked syntheses of quantitative and qualitative systematic review with involvement of CYP using the INVOLVE principles | NHS hospital trust | General mental health |

*IDD-MH* intellectual/developmental disabilities and co-occurring mental health conditions, *CBPR* community-based participatory research, *CYP* children and young people, *NHS* National Health Service in England

1. **Reporting assessment using the Quality of Reporting Involvement and Engagement of Patients and the Public Appraisal Tool (QRIPPAT) (Rouncefield-Swales et al., 2021)**

| **First author** | **Reports aim of involvement activity?** | **Reports term used to describe involvement?** | **Reports definition of term used?** | **Reports involved population demographics?** | **Reports conceptual models/frameworks?** | **Reports methods used to engage/involve?** | **Reports evaluation of involvement impact?** | **Reports impact of involvement?** | **Reports contextual/process factors?** | **Reports critical learning?** | **Score** |
| --- | --- | --- | --- | --- | --- | --- | --- | --- | --- | --- | --- |
| Anang 2019 | Y | Y | Y | S | Y | Y | N | Y | Y | Y | 8.5 |
| Babbage 2024 | Y | Y | Y | Y | Y | Y | Y | Y | Y | Y | 10 |
| Blueprint 2022 | Y | Y | Y | S | Y | Y | Y | Y | Y | Y | 9.5 |
| Cheng 2021 | Y | Y | S | Y | S | Y | N | Y | Y | Y | 7 |
| Culbong 2023 | Y | Y | Y | Y | Y | Y | S | Y | Y | Y | 9.5 |
| Dosso 2024 | Y | Y | N | S | N | Y | Y | Y | Y | Y | 7.5 |
| Figueroa 2025 | Y | Y | S | Y | Y | Y | Y | Y | Y | Y | 9.5 |
| Hargrove 2019 | Y | Y | Y | Y | Y | Y | Y | Y | Y | Y | 10 |
| Hetrick 2018 | Y | Y | Y | S | Y | Y | S | Y | Y | Y | 9 |
| Inge 2024 | S | Y | Y | S | Y | Y | Y | Y | Y | Y | 9 |
| Katapally 2020 | S | Y | Y | S | Y | Y | N | Y | Y | Y | 8 |
| Libon 2023 | S | Y | N | S | N | Y | N | Y | Y | Y | 6 |
| Liegghio 2013 | Y | Y | Y | Y | Y | Y | Y | Y | Y | Y | 10 |
| Mance 2010 | Y | Y | Y | Y | Y | Y | N | Y | Y | Y | 9 |
| Perez-Aronsson 2022 | S | Y | N | S | N | Y | Y | Y | Y | Y | 7 |
| Povey 2022 | Y | Y | Y | Y | Y | Y | N | Y | Y | Y | 9 |
| Povey 2020 | Y | Y | N | Y | N | Y | N | Y | Y | Y | 7 |
| Randall 2018 | Y | Y | Y | S | Y | Y | Y | Y | Y | Y | 9.5 |
| Rocha 2023 | Y | Y | Y | S | Y | Y | Y | Y | Y | Y | 9.5 |
| Savaglio 2025 | Y | Y | Y | Y | Y | Y | S | Y | Y | Y | 9.5 |
| Schwartz 2020 | Y | Y | N | S | N | Y | N | Y | Y | Y | 6.5 |
| Sheikhan 2021 | Y | Y | Y | N | Y | Y | Y | Y | Y | Y | 9 |
| Standley 2023 | Y | Y | Y | Y | Y | Y | S | Y | Y | Y | 9.5 |
| Stoyanov 2021 | Y | Y | N | Y | N | Y | N | Y | Y | Y | 7 |
| Thomson 2022 | Y | Y | Y | S | Y | Y | Y | Y | Y | Y | 9 |
| Viksveen 2022 | Y | Y | S | S | N | Y | Y | Y | Y | Y | 8 |
| Walker 2021 | Y | Y | Y | S | Y | Y | Y | Y | Y | Y | 9.5 |

Note: Y=Yes, N=No, S=Somewhat. Scores are calculated based on aggregating ratings across all questions with a Y scoring 1, N scoring 0 and S scoring 0.5

1. **PRISMA Checklist**

| **Section and Topic** | **Item #** | **Checklist item** | **Location where item is reported (Section title and paragraph number)** |
| --- | --- | --- | --- |
| **TITLE** | | |  |
| Title | 1 | Identify the report as a systematic review. | Title |
| **ABSTRACT** | | |  |
| Abstract | 2 | See the PRISMA 2020 for Abstracts checklist. | Abstract |
| **INTRODUCTION** | | |  |
| Rationale | 3 | Describe the rationale for the review in the context of existing knowledge. | Introduction paragraphs 5 & 6 |
| Objectives | 4 | Provide an explicit statement of the objective(s) or question(s) the review addresses. | Introduction paragraph 7 |
| **METHODS** | | |  |
| Eligibility criteria | 5 | Specify the inclusion and exclusion criteria for the review and how studies were grouped for the syntheses. | Eligibility criteria paragraph 1 and Table 1 |
| Information sources | 6 | Specify all databases, registers, websites, organisations, reference lists and other sources searched or consulted to identify studies. Specify the date when each source was last searched or consulted. | Search strategy paragraph 2 |
| Search strategy | 7 | Present the full search strategies for all databases, registers and websites, including any filters and limits used. | Supplementary file section 1 |
| Selection process | 8 | Specify the methods used to decide whether a study met the inclusion criteria of the review, including how many reviewers screened each record and each report retrieved, whether they worked independently, and if applicable, details of automation tools used in the process. | Study Selection paragraph 1 |
| Data collection process | 9 | Specify the methods used to collect data from reports, including how many reviewers collected data from each report, whether they worked independently, any processes for obtaining or confirming data from study investigators, and if applicable, details of automation tools used in the process. | Data extraction paragraph 1 |
| Data items | 10a | List and define all outcomes for which data were sought. Specify whether all results that were compatible with each outcome domain in each study were sought (e.g. for all measures, time points, analyses), and if not, the methods used to decide which results to collect. | Data extraction paragraph 1 |
|  | 10b | List and define all other variables for which data were sought (e.g. participant and intervention characteristics, funding sources). Describe any assumptions made about any missing or unclear information. | Data extraction paragraph 1 |
| Study risk of bias assessment | 11 | Specify the methods used to assess risk of bias in the included studies, including details of the tool(s) used, how many reviewers assessed each study and whether they worked independently, and if applicable, details of automation tools used in the process. | Quality appraisal paragraph 1 |
| Effect measures | 12 | Specify for each outcome the effect measure(s) (e.g. risk ratio, mean difference) used in the synthesis or presentation of results. | NA |
| Synthesis methods | 13a | Describe the processes used to decide which studies were eligible for each synthesis (e.g. tabulating the study intervention characteristics and comparing against the planned groups for each synthesis (item #5)). | Data Synthesis and analysis paragraph 3 |
|  | 13b | Describe any methods required to prepare the data for presentation or synthesis, such as handling of missing summary statistics, or data conversions. | Data Synthesis and analysis paragraphs 1 & 2 |
|  | 13c | Describe any methods used to tabulate or visually display results of individual studies and syntheses. | Data Synthesis and analysis paragraphs 1 & 2 |
|  | 13d | Describe any methods used to synthesize results and provide a rationale for the choice(s). If meta-analysis was performed, describe the model(s), method(s) to identify the presence and extent of statistical heterogeneity, and software package(s) used. | Data Synthesis and analysis paragraphs 1 & 2 |
|  | 13e | Describe any methods used to explore possible causes of heterogeneity among study results (e.g. subgroup analysis, meta-regression). | NA |
|  | 13f | Describe any sensitivity analyses conducted to assess robustness of the synthesized results. | NA |
| Reporting bias assessment | 14 | Describe any methods used to assess risk of bias due to missing results in a synthesis (arising from reporting biases). | NA |
| Certainty assessment | 15 | Describe any methods used to assess certainty (or confidence) in the body of evidence for an outcome. | NA |
| **RESULTS** | | |  |
| Study selection | 16a | Describe the results of the search and selection process, from the number of records identified in the search to the number of studies included in the review, ideally using a flow diagram. | Results paragraphs 1 & 2 plus figure 2 |
|  | 16b | Cite studies that might appear to meet the inclusion criteria, but which were excluded, and explain why they were excluded. | NA |
| Study characteristics | 17 | Cite each included study and present its characteristics. | Description of studies paragraph 1, Supplementary file section 3 |
| Risk of bias in studies | 18 | Present assessments of risk of bias for each included study. | Supplementary file 4 |
| Results of individual studies | 19 | For all outcomes, present, for each study: (a) summary statistics for each group (where appropriate) and (b) an effect estimate and its precision (e.g. confidence/credible interval), ideally using structured tables or plots. | NA |
| Results of syntheses | 20a | For each synthesis, briefly summarise the characteristics and risk of bias among contributing studies. | NA |
|  | 20b | Present results of all statistical syntheses conducted. If meta-analysis was done, present for each the summary estimate and its precision (e.g. confidence/credible interval) and measures of statistical heterogeneity. If comparing groups, describe the direction of the effect. | NA |
|  | 20c | Present results of all investigations of possible causes of heterogeneity among study results. | NA |
|  | 20d | Present results of all sensitivity analyses conducted to assess the robustness of the synthesized results. | NA |
| Reporting biases | 21 | Present assessments of risk of bias due to missing results (arising from reporting biases) for each synthesis assessed. | NA |
| Certainty of evidence | 22 | Present assessments of certainty (or confidence) in the body of evidence for each outcome assessed. | NA |
| **DISCUSSION** | | |  |
| Discussion | 23a | Provide a general interpretation of the results in the context of other evidence. | Discussion paragraphs 1- 8 |
|  | 23b | Discuss any limitations of the evidence included in the review. | Quality appraisal paragraph 1, Barriers and facilitators to involvement paragraph 1, Strengths and limitations |
|  | 23c | Discuss any limitations of the review processes used. | Strengths and limitations paragraph 4 |
|  | 23d | Discuss implications of the results for practice, policy, and future research. | Implications paragraphs 1 & 2 |
| **OTHER INFORMATION** | | |  |
| Registration and protocol | 24a | Provide registration information for the review, including register name and registration number, or state that the review was not registered. | Search strategy paragraph 1 |
|  | 24b | Indicate where the review protocol can be accessed, or state that a protocol was not prepared. | Search strategy paragraph 1 |
|  | 24c | Describe and explain any amendments to information provided at registration or in the protocol. | NA |
| Support | 25 | Describe sources of financial or non-financial support for the review, and the role of the funders or sponsors in the review. | TBC |
| Competing interests | 26 | Declare any competing interests of review authors. | TBC |
| Availability of data, code and other materials | 27 | Report which of the following are publicly available and where they can be found: template data collection forms; data extracted from included studies; data used for all analyses; analytic code; any other materials used in the review. | NA |

*From:*  Page MJ, McKenzie JE, Bossuyt PM, Boutron I, Hoffmann TC, Mulrow CD, et al. The PRISMA 2020 statement: an updated guideline for reporting systematic reviews. BMJ 2021;372:n71. doi: 10.1136/bmj.n71. This work is licensed under CC BY 4.0. To view a copy of this license, visit <https://creativecommons.org/licenses/by/4.0/>
